# The role of peer, parental, and school norms in predicting adolescents’ attitudes and behaviours of majority and different minority ethnic groups in Croatia

**DOI:** 10.1371/journal.pone.0227512

**Published:** 2020-01-10

**Authors:** Lana Pehar, Dinka Čorkalo Biruški, Tea Pavin Ivanec

**Affiliations:** 1 Department of Psychology, Faculty of Humanities and Social Sciences, University of Zagreb, Zagreb, Croatia; 2 Faculty of Teacher Education, University of Zagreb, Zagreb, Croatia; University of Pennsylvania, UNITED STATES

## Abstract

Social norms in general have an important role in the regulation of intergroup relations. However, the effects of one specific type of social norms–in-group norms about intergroup contact–have not yet been extensively studied, especially among groups of different status or in different intergroup contexts. The purpose of this research was to examine the effects of three types of contact norms (peer, parental and school) on four intergroup outcomes (in-group bias, social distance, tendency to discriminate, prosocial behaviour towards the outgroup) among ethnic majority and minority adolescents from four different intergroup contexts of the Republic of Croatia, as well as to test for moderating effects of age, social status and intergroup context in the strength of these effects. The research was carried out on a sample of 1440 elementary and high school students, members of Croatian majority, and Serbian, Hungarian, Czech, and Italian minority. The results indicated that although all three types of norms predict most of the intergroup outcomes, their relative importance depends on the specific type of intergroup outcome (attitudinal or behavioural), group social status (majority or minority), intergroup context (history of a recent intergroup conflict or not), and for peer norms on the age of the adolescent.

## Introduction

Social norms have long been considered important determinants of intergroup relations [1,2]. Some authors even argue that stereotypes and prejudices are predominantly the result of shared in-group norms [3,4]. The idea is based on the assumption that individuals belonging to subjectively meaningful social groups learn and share information about the appropriate treatment of relevant outgroups and that such information influences their intergroup attitudes and behaviours. A substantive body of research involving adults, children and adolescents has confirmed that in-group norms, as perceived by the group members, predict a wide range of intergroup outcomes, such as in-group favouritism [5], outgroup attitudes [6–8], quantity and quality of intergroup contact [9], as well as positive and negative intergroup behaviours [10,11]. However, in previous research, norms related to intergroup behaviours have been conceptualised in different ways, including norms about the expression or suppression of prejudices (e.g. [6,12,13]), norms regarding general attitudes towards outgroup members (e.g. [8,14]) and more recently norms about intergroup contacts (e.g. [15–18]). As intergroup contact in its various forms is one of the crucial factors determining the relationships between different social groups [19,20], the present study focuses on the content and effects of norms about intergroup contact. Because adolescence is a developmental period in which intergroup attitudes, as well as awareness of and conformity to in-group norms become increasingly important [21,22], we will explore normative influences on interethnic relations of adolescents.

### In-group norms about intergroup contact

In-group norms about intergroup contact can be thought of as perceptions of typicality or social approval of intergroup contact in one’s immediate social context. They provide information if and to what extent the in-group members associate with the outgroups, and if they approve and encourage positive intergroup interactions. This information is important because if people perceive that in-group members prohibit or avoid cross-group relations, their own relations with the outgroup are likely to be negatively affected. Conversely, the perception of positive norms should lead to better personal contact experiences, and consequently to more favourable intergroup outcomes [23]. Research focusing on broader in-group contact norms (i.e. norms held by the whole in-group or by a range of referents within the in-group) has shown that perceiving positive norms about intergroup contact is related to more intergroup empathy and trust [24], positive outgroup attitudes and expectancies for future contact [17,18], as well as decreased intergroup anxiety and threat ([25], Study 1). Although general group norms are undoubtedly important, people are usually influenced by several specific normative in-group sources operating in their proximal social environment. For children and adolescents, the most important influences come from their parents, peers and school.

#### Parental norms

Parents as primary socialisation agents influence intergroup attitudes of their children considerably (for a review, see [26]), and much of that influence is likely to be achieved through the perception of and adherence to parental norms. Parental intergroup behaviours (e.g. their own interactions with members of the outgroup) and other messages they send (e.g. explicit messages about acceptability of intergroup contact) can contribute to how their children perceive the norms about intergroup contact. Indeed, children’s reactions to parental norms have been widely studied, which is not the case with adolescents.

Nevertheless, there are findings indicating considerable influence of parental norms on adolescents’ intergroup attitudes. For example, Mähönen et al. [27] studied how perceived attitudes of family members towards immigrants relate to blatant and subtle immigrant attitudes of Finnish adolescents. They found that the perception of family norms regarding outgroup attitudes had a direct effect on adolescents’ attitudes, and a moderating effect on the relationship between quality of interethnic contact and subtle attitudes of boys. In addition, Edmonds and Killen [28] found a strong relationship between perceived parental norms about cross-race relationships and adolescents’ intergroup contact. Although they did not measure normative effects on adolescents’ attitudes, we know that outgroup attitudes are generally related to personal contact experiences [19]. In other words, it is possible that parental norms influence adolescents’ attitudes both directly and indirectly, by influencing their willingness to engage in intergroup contact. The proposed direct effect was supported by Ata et al. [15], who showed that perceived parental approval of intergroup contact was negatively related to adolescents’ social distance towards Muslims.

#### Peer norms

Children and adolescents are not influenced only by the norms conveyed by their parents. As they grow older, peers and friends become increasingly important factors in their social experiences. Indeed, research has shown that with transition from childhood to adolescence peer relations become more frequent and more salient, and adolescents attach great importance to perceived expectations and norms of their peer groups [29]. Perception of intergroup contacts of their peers and related norms the peers convey may influence adolescents’ relations with outgroup members regardless of their own attitudes towards intergroup contact. Unlike parental norms, effects of peer norms about intergroup contact have been studied more extensively. For example, De Tezanos-Pinto et. al. [16] found that perceiving norms against intergroup contacts among in-group friends is related to negative outgroup attitudes of adolescents. Furthermore, perceived peer norms supporting cross-group relations were also associated with higher perceived outgroup variability, both directly and through increased empathy towards the outgroup [30]. Finally, positive peer contact norms were also related to prosocial outgroup behaviour through increased quantity and quality of intergroup contact [31].

Although both peers and parents have significant roles in shaping adolescents’ behaviours in various domains, there is little research comparing their relative normative effects on intergroup attitudes and behaviours. Moreover, it has been suggested that the importance of parental and peer influences generally varies with the age of adolescent, though there are two contrasting views on the age-related changes in the strength of these influences. Due to the growing need for autonomy and increasing time adolescents spend with their peers, some authors have argued that peer influences strengthen in middle adolescence (i.e. 12 to 15 years), while parental influences decrease steadily with age [32]. Conversely, more recent research has shown that resistance to peer influence increases linearly throughout adolescence [33,34]. In one of the few studies examining simultaneously peer and family normative influences on intergroup attitudes during middle adolescence, Mähönen et al. [27] found that perceptions of family and peer attitudes towards immigrants have equally strong direct effects on intergroup attitudes of Swedish adolescents. In contrast, a longitudinal study of Miklikowska [35], which focused on actual rather than perceived attitudes of parents and peers, showed that parental self-reported attitudes predicted changes in anti-immigrant attitudes of Swedish youth in both early and mid-adolescence, while the effects of peers were limited to early adolescence, suggesting that their normative impact diminishes over time. However, to our knowledge, no research has examined which of the perceived norms about contact with the outgroup–those of parents or peers—have a stronger influence on intergroup attitudes and behaviours of adolescents and whether the strength of these influences differs depending on adolescents’ age.

#### School norms

In his influential contact hypothesis, Allport [1] proposed that support of intergroup contact from relevant authorities and institutions establishes a norm of acceptance, and thus increases the positive effects of intergroup contact on attitudes. The norms that adolescents perceive within their schools, as institutions in which they spend a significant amount of their time, may thus influence their intergroup attitudes and behaviours. If these influences are confirmed, school norms may have an especially important role in the development of positive intergroup relations, as rules, messages and values in the school context are generally more amenable to change then those in the peer and family environments.

The effects of perceived school norms have so far been mostly examined in relation to intergroup contact outcomes, and often in contrast to the effect of peer norms. For instance, Schachner and colleagues [36] found that positive contact norms in the classroom (i.e. perceived support for intergroup contact and cooperation from teachers and students) significantly predicted early adolescents’ intentions to socialise with the outgroup, as well as actual intergroup friendships. In a recent cross-sectional study comparing the effects of peer and school norms, Tropp et al. ([9], Study 1) showed that perceived norms supporting cross-ethnic relations among school staff and peers predicted greater interest and comfort in cross-ethnic relations, and higher contact quality among ethnic majority and minority students. Moreover, the effects of perceived peer norms were stronger than those of the school. However, in their longitudinal study perceived school contact norms predicted greater interest, comfort and quality of intergroup contact over time ([9], Study 2).

In addition to contact outcomes, experimental research with children suggests that intergroup attitudes can also be affected by perceived peer and school norms. For example, Nesdale and Lawson [37] observed that experimentally manipulated school and peer norms of inclusion lead to more positive attitudes towards outgroup members independently of each other. However, one of the few research studies looking at different roles of peer and school norms in explaining adolescents' intergroup behaviours, showed that perception of school norms about intergroup contact, unlike perception of peer norms, was not related to either positive or negative intergroup behaviours [31].

Although the studies so far did examine the relative influences of peer and school contact norms in adolescents of different ages, none of them have explicitly tested the potential age differences in the strength of those influences which could possibly explain inconsistencies in the findings in different studies. Moreover, the relative effects of three types of norms that are important for adolescents (parental, peer and school norms) are still unclear.

#### Normative effects in diverse social groups and intergroup contexts

Previous studies of in-group contact norms have focused primarily on normative effects among majority groups, thus neglecting how minority groups perceive and react to such norms. However, intergroup contact research has shown that the relationship between contact and intergroup attitudes depends on the social status of the group; the contact-attitude link is typically weaker for minority members [38]. It may be, as Tropp and Pettigrew [38] pointed out, that optimal contact conditions, including normative support for intergroup interactions, are perceived and their influence is exercised differently across the groups. Few studies that examined status differences in the effects of contact norms have produced mixed results.

First, some findings suggested that although majority and minority groups differ in their perception of contact norms, whereby minority perceives them to be less positive than the majority, their effects on intergroup outcomes are largely the same in both groups. For instance, Schachner et al. [36] reported that early adolescent immigrants in Germany perceived less support for intergroup contact and cooperation within their schools than their non-immigrant peers. However, there were no status differences in the strength of the association between school contact norms and intentions to socialise with the outgroup. Likewise, Gomez et al. [17] found equally strong effects of perceived in-group norms on intergroup attitudes and expectations for future contact among Spanish majority and immigrant minority adolescents.

Second, there are studies showing differences between majority and majority youth with respect to the effects of different types of perceived contact norms. For example, a longitudinal study among German and Turkish preadolescents attending ethnically heterogeneous schools showed that although perceived peer norms about cross-ethnic friendships predicted preference for the same-ethnic friendships over time in both groups, the effects of peer norms were particularly strong for the majority [39]. In addition, Feddes, Noack and Rutland [40] found that peer norms about intergroup friendships partially mediate the relationship between direct contact and outgroup evaluations only among ethnic majority children. Finally, Tropp et al. [9] provided both cross-sectional and longitudinal evidence of different patterns of peer and school norm effects among majority and minority adolescents in two different majority-minority contexts. However, majority-minority differences in the effect of parental norms have not yet been studied.

In addition, previous research on intergroup contact norms has mainly neglected the role of wider social context in which these norms operate, thus raising issues of ecological validity. Group norms are context dependent and influenced by specific in-group and intergroup dynamics of the groups involved [41]. According to the social identity perspective, an important contextual factor influencing social identities is the presence of intergroup conflict. Intergroup conflict is likely to make social identities contextually salient and heighten individual’s identification with the in-group [42]. In turn, stronger identification with a contextually relevant in-group enhances adherence to in-group norms [43]. In line with this rationale, it can be assumed that intergroup behaviours of in-group members will be more strongly influenced by in-group norms in contexts with a more recent or intense history of intergroup conflict than in those with relatively harmonious relations between the majority and minority. However, to our knowledge, only two previous studies have explored the effects of intergroup contact norms in two different intergroup contexts [9,25], and even those have used different norm measures across contexts, making contextual comparisons of normative influences difficult, if not impossible.

### The present research

The studies so far indicate that in-group norms about contact with the outgroup have an important role in determining a range of intergroup outcomes. However, these norms have been operationalised inconsistently and not comprehensively. For example, most studies did not consider several important sources of normative influence for adolescents separately. Some have combined items involving different sources of normative influence (e.g. best friends, friends, family, school, larger society, whole in-group) into a single measure [17,18], making it difficult to evaluate the unique effects of norms coming from specific reference groups. Others sought to assess the normative effects of a single reference group such as in-group friends [16,30], classmates [40,44], parents [15], and school authorities [36] or have differentiated the effects of up to two sources of influence [9,27,31]. Given that adolescents are often exposed to different, even conflicting norms simultaneously, it is important to determine sources of normative influence which are most likely to affect their intergroup attitudes and behaviours. However, to our knowledge, no research has simultaneously tested relative effects of norms conveyed by peers, parents and schools. Moreover, although some authors hypothesise that the pressure to conform to norms of specific reference groups changes with age of the adolescent, age differences in the strength of different types of normative influences are still unclear. Finally, research of in-group contact norms among majority and minority groups, and in different intergroup contexts has been limited. It is crucial to examine the norms about contact in diverse social groups and environments because members of majority and minority groups may differ in the way they perceive and react to contact norms and because different intergroup contexts may alter the way these norms shape majority-minority dynamics.

In order to address challenges we have pointed out, we conducted a study among majority and minority adolescents in four different intergroup contexts in the Republic of Croatia. In each context we focused on Croats as the majority group and a specific minority group which exercises the right to education in its mother tongue due to its high representation in the local population. These minority groups are Serbs, Hungarians, Czechs and Italians. These four groups practise the schooling of their children completely in their minority language. This practice (i.e. separate schools for minority students or separate minority classes within the same school as majority students) was a criterion for the selection of participants in the study. Besides these shared educational practices regulated as a minority educational right in the Republic of Croatia, the four majority-minority groups differ in their histories and in their current dynamics of intergroup relations.

#### Social context of the study

The Croatian-Serbian context includes the city of Vukovar and its surroundings, located in the Vukovar-Sirmium County in which Croats make up 79.2%, Serbs 15.5%, and other minorities 5.3% of the total population. These are post-conflict communities with a strong ethnic division between Croats and Serbs due to recent war in the 1990s. During the 1991–1995 war Vukovar suffered massive destruction and victimisation, and today also faces severe impoverishment and high unemployment rate [45]. Although Croatian and Serbian language, as well as many cultural traditions, are very similar, this intergroup context is considered as being ethnically divided, with only superficial contacts between the groups, and palpable interethnic tensions [46].

The Croatian-Hungarian context encompasses the Osijek-Baranja County with 85.9% Croats, 2.7% of Hungarians and 11.4% of other minorities. The Hungarian minority lives mostly in small, relatively ethnically homogenous villages, slightly separated from the Croatian majority. Hungarians and Croats lived in the same state for about 800 years, first in the unified kingdom and then within the Habsburg Empire and the last and only open conflict dated back to 1848. Though interethnic relations of Croats and Hungarians varied throughout history, they have been relatively positive and peaceful for a long time. However, some stereotypes related to historical antagonism and social distance between Croats and Hungarians still exist in Croatian society [47].

The Croatian-Czech context refers to the city of Daruvar and its surroundings located in Bjelovar-Bilogora County (84.8% Croats, 5.3% Czechs and 9.9% of other minorities). Czech minority has been present in this region for more than 150 years. They settled in Croatia during the 18th and 19th century when both Croatian and Czech territories were part of the Austrian-Hungarian Monarchy [48]. The Czechs and Croats have never had a history of intergroup conflict and their mutual relations in this region can be described as mostly harmonious. Due to their high level of integration in the local community, Czechs in this region even speak a unique form of the Czech idiom which is strongly influenced by Croatian language.

Finally, the Croatian-Italian context includes the Istria County (68.3% Croats, 6.0% Italians and 25.7% of other minorities), as one of the most ethnically mixed regions in Croatia. The history of conflict between Croats and Italians dated back after World War I, when almost all of the Istrian peninsula was annexed by Italy and exposed to forced Italianisation. After World War II, Istria was returned to the Croatian territory (under the jurisdiction of former Yugoslavia), and a significant percentage of Italians in Istria were forced to leave. Since that time the two groups have largely managed to develop mostly positive intergroup relations and a strong sense of regional identity. The rights of Italian minority are even protected by the Statute of the Istrian County which supports bilingualism and multicultural and multi-ethnic features of Istria.

#### Aim of the study

The purpose of the research was to examine the effects of three types of contact norms (peer, parental and school) on a variety of intergroup attitudes (in-group bias, social distance) and behavioural tendencies (tendency towards outgroup discrimination, prosocial behaviour towards the outgroup). We also wanted to test for moderating effects of adolescent’s age, social status and intergroup context in the strength of these effects. We predicted that all three types of norms will be related to all intergroup outcomes. More specifically we predicted that perception of more positive peer, parental and school norms about the contact with the outgroup will predict lower levels of intergroup bias, social distance and tendency to discriminate the outgroup, and higher levels of prosocial behaviour towards the outgroup. In line with more recent findings on the age-related changes in the peer and parental normative pressures, we predicted that the effects of peer contact norms on intergroup attitudes and behaviours will diminish with adolescent’s age, while the effects of parental norms will not be moderated by age. Due to the previous mixed findings regarding the role of group status as a potential moderator of these associations, we did not make any direct hypothesis about strength of normative effects among majority and minority adolescents. However, because norm adherence strengthens in conflictual settings, we expected stronger effects of contact norms on outgroup attitudes and behaviours in contexts with a more recent history of intergroup conflict.

## Materials and methods

### Procedure and participants

Data collection was carried out during spring and autumn of 2017 in four intergroup contexts where minority children attend schools in their mother tongue. All research procedures were approved by the institutional review board at the Department of Psychology, University of Zagreb. Participants were recruited with the help of school staff in 21 elementary (4 with majority, 7 with minority and 10 with both majority and minority language of instruction) and 10 high schools (4 with majority, 3 with minority and 3 with both majority and minority language of instruction). In most schools, we recruited students attending either 6^th^ and 7^th^ grade of elementary or 2^nd^ and 3^rd^ grade of high school, except for a few schools in which we also recruited students from 8^th^ grade of elementary or 1^st^ and 4^th^ grade of high school to compensate for the lack of minority students in the sample. Only students with written parental consent (for children under 14 years) or student assent were included in the sample. The research team administered questionnaires during one class period (45 min). The questionnaires for all minority participants, except the Czech (who preferred to fill out the questionnaire in Croatian language), were translated into their mother tongue by a professional translator and with supervision of psychologists who were fluent in the respective minority language. In total, 1440 adolescents completed the questionnaire (50% minority, 55% female) and their mean age was 15.27 years (*SD* = 1.98) (*M* = 13.48, *SD* = 0.95 for elementary students; *M* = 16.87, *SD* = 1.09 for high school students). Out of the total sample, 563 (39%) adolescents were recruited from the Croatian-Serbian context (of those 58% were majority adolescents), 294 (20%) from the Croatian-Czech context (53% majority), 170 (12%) from the Croatian-Hungarian context (43% majority), and 413 (29%) from the Croatian-Italian context (40% majority). The composition of the total sample per intergroup context, language of instruction and type of school is presented in Table 1.

**Table 1:** Sample composition.

Context	Language of instruction	Elementary school *n*	High school *n*	Total *n*
Croatian-Serbian context	Majority	135	190	325
	Minority	97	141	238
Croatian-Hungarian context	Majority	29	44	73
	Minority	63	34	97
Croatian-Czech context	Majority	110	47	157
	Minority	114	23	137
Croatian-Italian context	Majority	53	110	163
	Minority	103	147	250
Total		704	736	1440

### Measures

All measures used in the questionnaire were contextually adapted, with items always describing the same content but considering the relevant majority-minority relationship. For example, within the Croatian-Serbian context, Croatian students responded to items assessing their attitudes or behaviours towards their Serbian peers, and Serbian students towards their Croatian peers. Furthermore, in the Croatian-Italian context, Croatian students responded towards their Italian peers and vice versa, etc.

#### In-group norms about intergroup contact

Three subscales for measuring peer, parental and school norms about intergroup contact were developed based on a previous qualitative study conducted in 20 of the previously described schools, as well as existing scales of contact norms. Based on the content analysis of 26 focus group discussions with majority and minority students, 15 items describing adolescents’ perceptions of contact norms were formulated. Participants assessed if they agree with each item on a 4-point Likert scale ranging from 1 (completely disagree) to 4 (completely agree), with higher values indicating more positive norms about intergroup contact.

Confirmatory factor analysis using maximum likelihood estimation with robust standard errors was conducted in R lavaan package [49] to evaluate the factor structure underlying these 15 items. We expected items designed to measure peer, parental and school contact norms to load onto their respective latent factors (5 items on each type of norm). To assess the overall model fit, we used the Satorra-Bentler scaled chi-square test (S-B χ^2^), the comparative fit index (CFI), the root mean square of approximation (RMSEA), and the standardised root mean square residual (SRMR). In line with literature recommendations, non-significant S-B χ^2^ (although significant values are acceptable when the sample size is large), CFI ≥ .95, SRMR ≤ .08, and RMSEA ≤ .06 were considered indicative of a good model fit [50]. The three-factor model including all 15 items produced a poor fit (S-B χ^2^ (87, N = 1175) = 798.185, p < .001; CFI = .82; RMSEA = .08; SRMR = .08). In the next step, we revised the model iteratively by removing items with low factor loadings and/or problematic wording one at a time, until a satisfactory model fit was achieved (S-B χ^2^ (11, N = 1284) = 80.052, p < .001; CFI = .97; RMSEA = .07; SRMR = .04). This model included two items measuring peer norms (“My friends usually go out with peers of Croatian/Serbian/Hungarian/ Czech/Italian nationality”, “Romantic relationships between my peers of Croatian/Serbian/ Hungarian/Czech/Italian nationality are common”), two items measuring parental norms (“My parents hang out with individuals of Croatian/Serbian/Hungarian/Czech/Italian nationality”, “My parents have close friends of Croatian/Serbian/Hungarian/Czech/Italian nationality”) and three items measuring school norms regarding intergroup contact (“In my school it is common that students of Croatian/Serbian/Hungarian/Czech/Italian nationality hang out with each other”, “In my school all students are included in different activities, regardless of their nationality”, “Our teachers encourage us to socialise with our peers of Croatian/Serbian/Hungarian/Czech/Italian nationality”). Standardised factor saturations of the seven items ranged from .50 to .86.

Cronbach's alphas on the total sample was .60 for the peer norms subscale (.66 for majority subsample, .46 for minority subsample, .60 for Croatian-Serbian and Croatian-Hungarian subsample, .52 for Croatian-Czech subsample, and 0.49 for Croatian-Italian subsample), .86 for the parental norms subscale (.89 for majority subsample, 0.78 for minority subsample, .87 for Croatian-Serbian and Croatian-Hungarian subsample and .82 for Croatian-Czech, and Croatian-Italian subsample), and .64 for the school norms subscale (.61 for majority subsample, .67 for minority subsample, .68 for Croatian-Serbian subsample, .55 for Croatian-Hungarian subsample, .54 for Croatian-Czech subsample, and .50 for Croatian-Italian subsample). Although Cronbach’s alphas for peer and school norms subscale are relatively low and vary across different subsamples, it should be noted that these two subscales consist of only two or three items, their Cronbach’s alphas are calculated in subsamples of different sizes and heterogeneity (e.g. minority subsamples have lower variability of total scores on the peer norm subscale than majority subsamples) and their total scores are non-normally distributed. Given that Cronbach’s alpha is strongly affected by the length of the scale (the more items in a scale the higher the reliability), depends on the sample being tested (the more heterogenous the sample, the larger the variance of the total scores and the higher the reliability) [51], and is sensitive to even minor deviations from normality [52], we deemed the reported coefficient alphas as adequate for the purpose of the research. In addition, Cronbach's alpha also depends on the content homogeneity of the items and the construct that is being assessed, and even unidimensional constructs can be conceptualised as having a number of different aspects [51]. The items on all norm subscales were formulated in a way that captures all relevant normative aspects that were identified during the qualitative study. However, factor analysis of the items in the quantitative study resulted in three subscales with different degree of content heterogeneity between the items. Specifically, parental norm subscale consists of two items with highly similar content (associated with parents’ close friendships and less intimate contacts) which resulted in higher reliability estimates than for peer and school norm subscales whose items tap into somewhat different normative aspects (e.g. frequency of contact and romantic relationships between members of different groups).

#### Tendency to discriminate against the outgroup

The tendency to discriminate against the outgroup was measured with the adapted version of tendency towards outgroup discrimination scale [53]. The scale consists of eight items describing the tendency of ethnic discrimination in everyday situations (e.g. “If I forgot to write my homework, I would rather get an F than copy it from a Croat/Serb/Hungarian/ Czech/Italian)”. By answering yes or no participants indicate whether they would necessarily choose a member from their own group in the described situation. The total score is expressed as the sum of all positive responses, with higher scores indicating greater intention to discriminate against the outgroup. Cronbach's alphas on the total sample was .80 (.82 for majority subsample, .77 for minority subsample, .81 for Croatian-Serbian subsample, .80 for Croatian-Hungarian subsample, .77 for Croatian-Czech subsample, and .79 for Croatian-Italian subsample).

#### Prosocial behaviour towards the outgroup

Prosocial behaviour towards members of the outgroup was assessed with the scale of active bystandership [54] which measures participants’ readiness for prosocial action towards a member of the outgroup in a situation where a member of their own group is a source of threat, hostility or aggression. The scale consists of five items describing different situations in which the members of one’s own group behave negatively towards the outgroup members (e.g. “When my peers insult students of Croatian/Serbian/Hungarian/Czech/Italian nationality”), where participants have to choose one response that best describes their reaction in a given situation (1 - “I support them or join them”, 2 - “I pretend not to notice/I ignore them”, 3 - “I ask them to stop”, 4 - “I ask my peers to help me stop it”). The total score is expressed as the mean of all item scores, with higher results indicating more prosocial behaviour towards the outgroup member. Cronbach's alphas on the total sample was .86 (.89 for majority subsample, .82 for minority sample, .87 for Croatian-Serbian subsample, .80 for Croatian-Hungarian subsample, .83 for Croatian-Czech subsample, and .85 for Croatian-Italian subsample).

#### Social distance towards the outgroup

Social distance was assessed with a modified version of the Bogardus social distance scale [55] which measures the degree of intimacy the individual is willing to accept with members of social outgroups. The scale consists of seven degrees of intimacy (“to live together in Croatia”, “to go to the same class”, “to live as a next-door neighbour“, “to be friends and socialise outside your home”, “to be friends who visit each other at home”, “to be relatives”, “to have him/her as a boyfriend/girlfriend”). Total score is expressed as the sum of all the degrees of intimacy participants are willing to accept, and higher score indicates more distance towards the outgroup. Cronbach's alphas on the total sample was .84 (.87 for majority subsample, .76 for minority subsample, .84 for Croatian-Serbian subsample, .81 for Croatian-Hungarian subsample, .77 for Croatian-Czech subsample, and .87 for Croatian-Italian subsample).

#### In-group bias

In-group bias was assessed with a measure of in-group bias [53] consisting of two separate evaluation continuums on which participants evaluate their general attitude towards their own and contextually relevant ethnic outgroup. Each continuum has 11 points, ranging from 0 (negative attitude) to 10 (positive attitude). Total score is expressed as difference between the in-group evaluation and outgroup evaluation, with higher scores indicating greater in-group bias.

## Results

We first examined status and contextual differences, as well as correlations of predictor and criterion variables using multivariate analyses of variance (MANOVAs), analyses of variance (ANOVAs), and bivariate correlations. In order to test the hypothesis, we conducted a series of path models in a single and multiple-group framework using R package lavaan [49]. Path models were fitted using maximum likelihood estimation with robust standard errors for continuous variables to account for deviations from multivariate normal distribution and heteroscedasticity in the data. All variables included in the path analysis were observed variables. All analyses were conducted with participants having no missing data (N = 1106).

Although all continuous variables had missing values, ranging from 49 (3.4%) for age to 106 (7.4%) for social distance, we found no systematic pattern of nonresponses across the study variables. In order to determine whether data were missing completely at random, we examined if the missing data on criterion variables are related to observed values of predictor variables using R package finalfit. Comparisons of cases with incomplete and complete data on in-group bias variable revealed no significant differences in mean values of peer (*p* = .699), parental (*p* = .216), and school norms (*p* = .152) variables. Comparisons on discrimination tendencies variable revealed no significant differences in mean values of peer (*p* = .907), parental (*p* = .125), and school norms (*p* = .161) variables. Comparisons on prosocial behaviour variable revealed no significant differences in mean values of peer (*p* = .518), parental (*p* = .976), and school norms (*p* = .799) variables. Finally, comparisons on social distance variable also revealed no significant differences in mean values of peer (*p* = .100), parental (*p* = .132), and school norms (*p* = .255) variables, showing that the probability of missing data on dependent variables is unrelated to the predictor variables. In addition, we performed the nonparametric test of homoscedasticity developed by Jamshidian and Jalal [56] which examines whether non-normally distributed data has values missing completely at random by testing for homogeneity of covariances between subsets of data having different patterns of missingness (including the one with no missing values). Results of the nonparametric test indicated that there is no sufficient evidence to reject the assumption of data missing completely at random (*T* = 8.192, *p* = .570). According to guidelines for handling missing data [57], if the data are consistent with the assumption of missing completely at random and the remaining sample after listwise deletion is large enough for adequate power (*N* ≥ 1000), then analysis can be performed on a complete sample containing no missing data.

### Descriptive statistics of the study variables

Table 2 presents means and standard deviations of all study variables for the total sample, majority and minority subsamples regardless of the context and subsamples from each of the four contexts regardless of participant’s group status. Two MANOVAs were performed on combined predictor and criterion variables to examine mean differences by group status and intergroup context. Significant group differences were found for both majority/minority status (*F*(6, 1099) = 44.13, *p* < .001, λ = .81, η^2^ = .19) and context (*F*(18, 3103) = 16.79, *p* < .001, λ = .77, η^2^ = .08). Follow-up ANOVAs revealed significant differences between majority and minority subsamples on peer norms (*F*(1, 1104) = 81.31, *p* < .001, η^2^ = .07), parental norms (*F*(1, 1104) = 141.40, *p* < .001, η^2^ = .11), discrimination tendencies (*F*(1, 1104) = 5.39, *p* = .021, η^2^ = .01), social distance (*F*(1, 1104) = 65.62, *p* < .001, η^2^ = .06), and in-group bias (*F*(1, 1104) = 146.30, *p* < .001, η^2^ = .12). Minority participants reported slightly higher levels of discrimination tendencies, lower levels of social distance and in-group bias, and perceived more positive peer and parental norms towards contact with the outgroup than majority participants.

**Table 2:** Means and standard deviations of main study variables for total sample and per status and contextual subgroups.

	Total sample (*n =* 1106)		Total Majority sample (*n = 543*)		Total Minority sample (*n = 563*)		Croatian-Serbian context (*n = 418*)		Croatian-Hungarian context (*n = 122*)		Croatian-Czech context (*n = 222*)		Croatian-Italian context (*n = 344*)	
Variable	*M*	*SD*	*M*	*SD*	*M*	*SD*	*M*	*SD*	*M*	*SD*	*M*	*SD*	*M*	*SD*
Peer norms	3.02	0.79	2.81	0.83	3.22	0.69	2.71	0.81	2.89	0.81	3.31	0.69	3.24	0.67
Parental norms	3.21	0.90	2.90	0.98	3.51	0.69	2.91	0.94	2.99	0.98	3.58	0.69	3.41	0.79
School norms	3.33	0.65	3.33	0.63	3.33	0.68	3.08	0.72	3.27	0.68	3.66	0.44	3.44	0.53
Discrimination tendencies	1.05	1.69	0.93	1.67	1.17	1.70	1.24	1.84	1.21	1.85	0.82	1.33	0.90	1.61
Prosocial behaviour	2.96	0.65	2.93	0.69	2.98	0.61	2.73	0.68	3.00	0.58	3.24	0.56	3.03	0.60
Social distance	0.99	1.71	1.40	2.04	0.59	1.19	1.54	1.99	1.01	1.68	0.64	1.34	0.53	1.34
In-group bias	0.90	2.82	1.89	2.67	-0.04	2.64	1.89	3.20	1.35	2.76	0.05	1.89	0.10	2.42
Age	15.40	1.97	15.47	1.96	15.33	1.97	15.22	1.89	15.69	1.91	14.70	1.94	15.97	1.93

One-way ANOVAs for contextual differences were followed by post hoc comparisons using Tukey's honestly significant difference test. Significant differences between intergroup contexts were found for all variables: peer (*F*(3, 1102) = 46.37, *p* < .001, η^2^ = .11), parental (*F*(3, 1102) = 40.00, *p* < .001, η^2^ = .10), and school norms (*F*(3, 1102) = 49.25, *p* < .001, η^2^ = .12), discrimination tendencies (*F*(3, 1102) = 4.40, *p* = .004, η^2^ = .01), prosocial behaviour (*F*(3, 1102) = 35.24, *p* < .001, η^2^ = .09), social distance (*F*(3, 1102) = 27.94, *p* < .001, η^2^ = .07) and in-group bias (*F*(3, 1102) = 37.42, *p* < .001, η^2^ = .09). Participants from the Croatian-Serbian and Croatian-Hungarian context perceived less positive peer and parental norms and reported more in-group bias than participants from the Croatian-Czech and Croatian-Italian context (all *p*s < .001). Participants from all intergroup contexts differed in their perception of school norms (all *p*s < .05); those from the Croatian-Serbian context perceived the least positive norms, followed by participants from the Croatian-Hungarian context, then the Croatian-Italian, and finally from the Croatian-Czech context. Participants from the Croatian-Serbian context reported higher levels of discrimination tendencies than those from the Croatian-Czech (*p* = .015) and Croatian-Italian context (*p* = .030). Participants from the Croatian-Serbian context reported the lowest levels of prosocial behaviour compared to all other contexts (all *p*s < .001), while participants from the Croatian-Czech context reported higher levels of prosocial behaviour compared to the Croatian-Hungarian and Croatian-Italian context (all *p*s < .01). Participants from the Croatian-Serbian context reported the highest levels of social distance compared to all other contexts (all *p*s < .01), while participants from the Croatian-Hungarian context reported higher levels than participants from the Croatian-Italian context (*p* = .029).

In addition, two one-way ANOVAs were performed to examine age differences between participants of majority and minority status and in different intergroup contexts. The results revealed non-significant age differences between majority and minority (*F*(1, 1104) = 1.39, *p* = .239, η^2^ = .00), but there were significant differences among four contexts (*F*(3, 1102) = 22.13, *p* < .001, η^2^ = .06). Participants from Croatian-Czech context were on average younger than participants from all other contexts (all *p*s < .01), and those from the Croatian-Serbian context were younger than those from the Croatian-Italian context (*p* < .001).

In Table 3 we present bivariate correlations among all variables for the total sample, majority and minority subsamples and contextual subsamples. As for the total sample, all correlations except those with age, were significant and ranged from low to moderate. Peer, parental and school norms were negatively related to discrimination tendencies, social distance and in-group bias, and positively related to prosocial behaviour. Similar patterns of correlations appeared within both majority and minority subsamples, with the exception of social distance and in-group bias which correlated more strongly with peer and parental norms among the majority. The pattern of correlations showed some differences between intergroup contexts. Peer and parental norms had higher correlations with negative indicators of intergroup relations in the Croatian-Serbian context than in other intergroup contexts.

**Table 3:** Correlations among key study variables for total sample and per status and contextual subgroups.

Variable	1	2	3	4	5	6	7
**Total (*n =* 1106)**							
1. Peer norms							
2. Parental norms	.50***						
3. School norms	.39***	.31***					
4. Discrimination tendencies	-.20***	-.20***	-.16***				
5. Prosocial behaviour	.28***	.29***	.29***	-.23***			
6. Social distance	-.38***	-.41***	-.19***	.46***	-.28***		
7. In-group bias	-.32***	-.38***	-.17***	.21***	-.28***	.47***	
8. Age	-.01	-.15***	-.24***	-.13***	-.22***	-.07*	.03
**Majority (*n =* 543)**							
1. Peer norms							
2. Parental norms	.47***						
3. School norms	.43***	.31***					
4. Discrimination tendencies	-.28***	-.27***	-.15**				
5. Prosocial behaviour	.25***	.31***	.31***	-.29***			
6. Social distance	-.37***	-.40***	-.15**	.59***	-.29***		
7. In-group bias	-.34***	-.40***	-.21***	.49***	-.38***	.56***	
8. Age	-.03	-.18***	-.24***	-.14**	-.29***	-.10*	-.05
**Minority (*n =* 563)**							
1. Peer norms							
2. Parental norms	.43**						
3. School norms	.37***	.36***					
4. Discrimination tendencies	-.17***	-.19***	-.17***				
5. Prosocial behaviour	.31***	.27***	.27***	-.17***			
6. Social distance	-.27***	-.25***	-.28***	.37***	-.28***		
7. In-group bias	-.15***	-.17***	-.15***	.03	-.18***	.26***	
8. Age	.03	-.10*	-.23***	-.11**	-.15***	-.06	.09*
**Croatian-Serbian context (*n =* 418)**							
1. Peer norms							
2. Parental norms	.40***						
3. School norms	.25***	.19***					
4. Discrimination tendencies	-.31***	-.37***	-.19***				
5. Prosocial behaviour	.25***	.25***	.19***	-.35***			
6. Social distance	-.36***	-.49***	-.14**	.65***	-.32***		
7. In-group bias	-.30***	-.39***	-.09	.39***	-.35***	.56***	
8. Age	-.07	-.18***	-.47***	.04	-.24***	.06	.07
**Croatian-Hungarian context (*n =* 122)**							
1. Peer norms							
2. Parental norms	.59***						
3. School norms	.45***	.37***					
4. Discrimination tendencies	.10	.04	-.04				
5. Prosocial behaviour	.19*	.22*	.21*	-.33***			
6. Social distance	-.14	-.17	-.05	.18	-.34***		
7. In-group bias	-.20*	-.22*	-.14	.11	-.30***	.30***	
8. Age	-.29***	-.34***	-.25**	-.19*	-.24**	-.02	.20*
**Croatian-Czech context (*n =* 222)**							
1. Peer norms							
2. Parental norms	.35***						
3. School norms	.45***	.31***					
4. Discrimination tendencies	-.09	.03	-.15*				
5. Prosocial behaviour	.15*	.11	.26***	.03			
6. Social distance	-.36***	-.21**	-.24***	.37***	-.05		
7. In-group bias	-.09	-.23***	-.09	-.02	.06	.15*	
8. Age	.19**	-.03	.04	-.37***	-.18**	-.24***	.00
**Croatian-Italian context (*n =* 344)**							
1. Peer norms							
2. Parental norms	.49***						
3. School norms	.29***	.19***					
4. Discrimination tendencies	-.12*	-.04	-.05				
5. Prosocial behaviour	.18***	.25***	.23***	-.06			
6. Social distance	-.30***	-.27***	-.05	.23***	-.09		
7. In-group bias	-.23***	-.26***	-.02	-.05	-.08	.34**	
8. Age	.02	-.13*	-.05	-.21***	-.26***	-.13*	-.02

* *p* < .05

** *p* < .01.

*** *p* < .001.

### Path model of normative effects on intergroup outcomes for the total sample

We first tested a path model using the total sample in which perceived peer, parental and school norms predicted in-group bias, social distance, discrimination tendencies and prosocial behaviour towards the outgroup. Given that our model did not include all relevant predictors of specified intergroup outcomes, residual variances of the four criterion variables were allowed to correlate with one another. As this model was fully saturated, we do not report fit index values, but instead focussed on the magnitude of its unstandardised parameter estimates.

**Fig 1:**
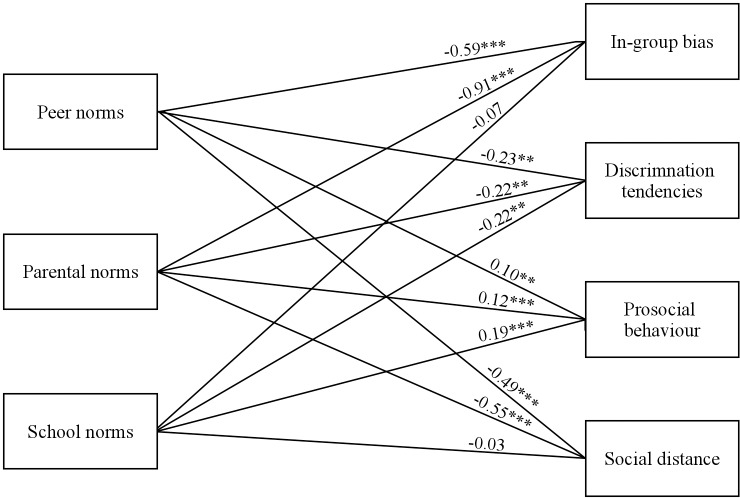
Path model of normative effects on intergroup outcomes for the total sample. Predictor path estimates are unstandardised regression coefficients (b). ** *p* < 0.01, *** *p* < 0.001.

Peer (*b* = -0.59, *SE* = 0.12, *p* < .001) and parental norms (*b* = -0.91, *SE* = 0.11, *p* < .001) were negatively related to in-group bias, but the effect of school norms was not significant (*b* = -0.07, *SE* = 0.16, *p* = .673). Peer (*b* = -0.23, *SE* = 0.08, *p* = .004), parental (*b* = -0.22, *SE* = 0.07, *p* = .002), and school norms (*b* = -0.22, *SE* = 0.09, *p* = .018) were negatively related to discrimination tendencies. Peer (*b* = 0.10, *SE* = 0.03, *p* = .001), parental (*b* = 0.12, *SE* = 0.03, *p* < .001), and school norms (*b* = 0.19, *SE* = 0.03, *p* < .001) were positively related to prosocial behaviour. Finally, peer (*b* = -0.49, *SE* = 0.08, *p* < .001) and parental norms (*b* = -0.55, *SE* = 0.08, *p* < .001) were also negatively related to social distance, while the effect of school norms was not significant (*b* = -0.03, *SE* = 0.08, *p* = .676).The model with estimated unstandardised regression parameters is shown in Fig 1. The normative set of predictors explained 17% of variance in in-group bias, 6% in discrimination tendencies, 14% in prosocial behaviour, and 20% of variance in social distance. As hypothesised, perception of positive peer, parental and school norms predicted higher levels of prosocial behaviour and lower levels of discrimination tendencies towards the outgroup. However, perception of positive peer and parental, but not school norms, predicted a lower level of in-group bias and social distance.

### Moderation analyses of normative effects on intergroup outcomes

The same theoretical model including norms as predictors of four intergroup outcomes was then tested using several moderated path analyses in SEM framework to examine whether adolescent’s age, social status and intergroup context moderate the effects of contact norms. Because age is a continuous predictor, the first moderation analysis was conducted through a path model which included mean centred norm predictors and age, as well as their interaction terms. Significant interactions terms were followed by a comparison of simple slopes to allow for the interpretation of the interaction effect. This model was also fully saturated, so we do not report fit index values. The moderating role of group status and intergroup context were analysed using separate multiple-group path analyses which allow for comparisons of regression coefficients across groups defined by a categorical moderator. Because we initially assumed that the groups might display different normative effects, we first assessed the overall moderation effect by comparing the fit of two nested models: an unconstrained model in which all regression paths were allowed to vary across the groups and a constrained model where all paths were set to be equal between the groups. Due to violation of the multivariate normality assumption, the models’ goodness of fit was assessed based on the Satorra-Bentler scaled chi-squared statistic (S-B χ^2^) and these two models were compared using a scaled Satorra-Bentler chi-square difference test (ΔS-B χ^2^ with an α-level of .05) because the use of S-B χ^2^ as a measure of fit also requires an adjustment to the chi-square difference test [58] If the test was significant, we next identified specific paths that differ between the groups by estimating a series of models in which we systematically constrained each regression path, one at a time, to be equal across the groups. If constraining a path resulted in a significant decrease in the model fit compared to the unconstrained model, this path was allowed to vary between the groups in the final model. This process enabled us to establish a partially constrained model specifying all paths that do and do not differ significantly across the groups.

#### Moderation effects of age

Moderated path analysis for age revealed significant negative effects of peer (*b* = -0.58, *SE* = 0.13, *p* < .001) and parental norms (*b* = -0.91, *SE* = 0.11, *p* < .001) on in-group bias. However, the effects of school norms (*b* = -0.02, *SE* = 0.16, *p* = .923), age (*b* = -0.01, *SE* = 0.04, *p* = .704) and of all interaction terms between norms and age were non-significant (all *p*s > .05). Age (*b* = -0.16, *SE* = 0.02, *p* < .001), peer (*b* = -0.17, *SE* = 0.08, *p* = .028), parental (*b* = -0.25, *SE* = 0.07, *p* < .001), and school norms (*b* = -0.38, *SE* = 0.09, *p* < .001) were negatively related to discrimination tendencies, again without any significant interaction (all *p*s > .05). Age (*b* = -0.05, *SE* = 0.01, *p* < .001), peer (*b* = 0.12, *SE* = 0.03, *p* < .001), parental (*b* = 0.10, *SE* = 0.03, *p* < .001), and school norms (*b* = 0.15, *SE* = 0.03, *p* < .001) had significant effects on prosocial behaviour, but none of the interaction terms between age and norms were significant (all *p*s > .05). Finally, age (*b* = -0.12, *SE* = 0.02, *p* < .001), peer (*b* = -0.45, *SE* = 0.08, *p* < .001) and parental norms (*b* = -0.59, *SE* = 0.07, *p* < .001), as well as the interaction between age and peer norms (*b* = 0.08, *SE* = 0.04, *p* = .026) significantly predicted social distance. However, the effects of school norms (*b* = 0.15, *SE* = 0.08, *p* = .074) and two other interaction terms were non-significant (all *p*s > .05).

**Fig 2:**
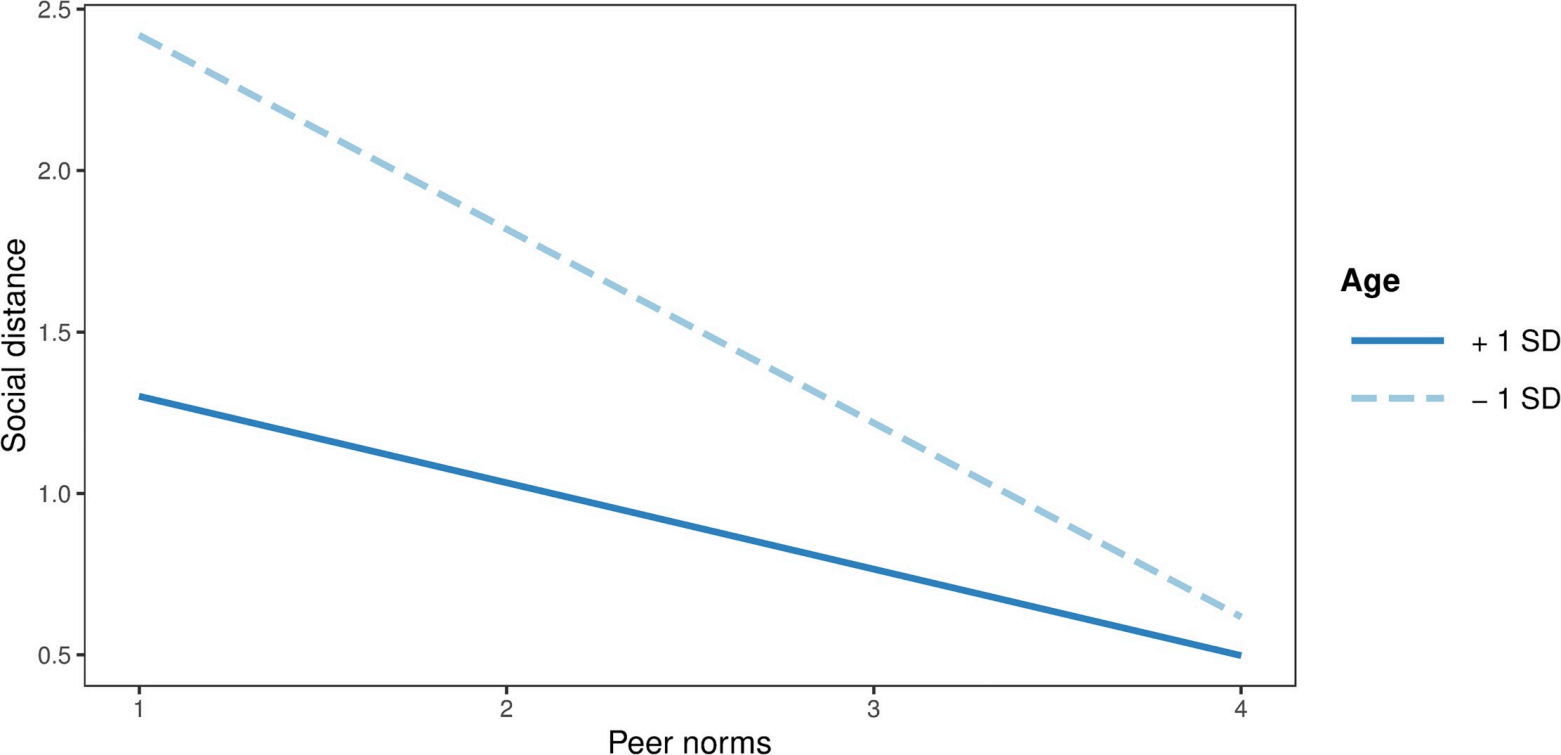
Interaction plot between peer norms and age on social distance.

Simple slopes for the association between peer norms and social distance were tested for low (-1 *SD* below the mean) and high (+1 *SD* above the mean) levels of age. Simple slopes revealed that the negative effect of peer norms on social distance was stronger among younger (*b* = -0.60, *SE* = 0.09, *p* < .001) than among older participants (*b* = -0.27, *SE* = 0.11, *p* = .01). Additional Johnson-Neyman analysis revealed that the negative relationship between peer norms and social distance became statistically non-significant at the age of 18 (*b* = -0.21, *SE* = 0.12, *p* = .080) In other words, perception of positive peer norms predicted lower levels of social distance towards the outgroup, but this effect diminished with adolescents’ age. Interaction effect of peer norms and age on social distance in shown graphically in Fig 2. The model including age, peer, parental and school norms, as well as their interactions, explained 17% of variance in in-group bias, 16% in prosocial behaviour, 9% in discrimination tendencies and 22% of variance in social distance.

#### Moderation effect of group status

Multi-group path analysis for status tested the hypothesised path model on separate samples of majority and minority participants. The unconstrained path model in which all paths were freed to vary across the status groups was fully saturated, so no fit statistics could be computed. However, the fit of this model differed significantly compared to the constrained model where all normative path coefficients were set equal between the groups (Δχ^2^(12) = 38.741, *p* < .001), indicating an overall moderation effect of group status. Further testing of group differences between specific normative regression paths revealed that the path from peer norms to prosocial behaviour, and paths from peer, parental and school norms to social distance differed significantly between majority and minority participants. The final partially constrained model in which only these four normative paths were allowed to vary across groups provided good overall fit and did not have a significantly different fit compared with unconstrained model (Δχ^2^(8) = 12.375, *p* = .135). Fit indices for the unconstrained, partially constrained and constrained model are reported in Table 4.

**Table 4:** Summary of fit statistics for the hypothesised multiple-group path model for group status.

Model	S-B χ^2^	*df*	*p*	CFI	RMSEA	SRMR
Unconstrained	-	-	-	-	-	-
Partially constrained	12.375	8	.135	.99	.04	.03
Constrained	38.741	12	.000	.98	.07	.04

**Fig 3:**
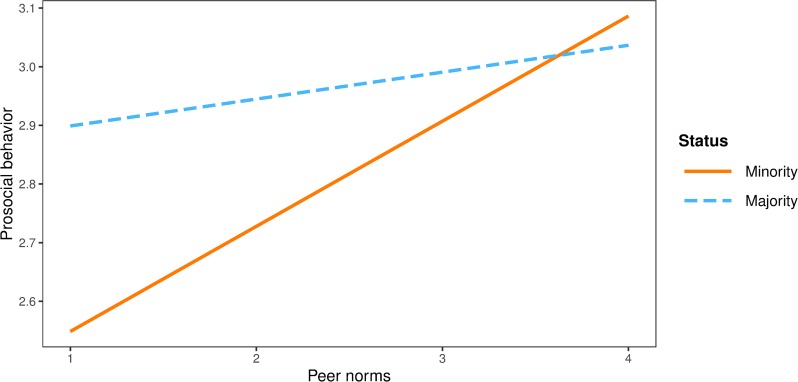
Interaction plot between peer norms and group status on prosocial behaviour.

In this final model, peer (*b* = -0.44, *SE* = 0.12, *p* < .001) and parental (*b* = -0.70, *SE* = 0.11, *p* < .001) norms had equal negative effects on in-group bias among both majority and minority participants, while the effect of school norms was not significant (*b* = -0.24, *SE* = 0.15, *p* = .119). The same negative effects of peer (*b* = -0.28, *SE* = 0.08, *p* < .001) and parental norms (*b* = -0.28, *SE* = 0.07, *p* < .001) on discrimination tendencies were found across subgroups, while the effect of school norms was not significant (*b* = -0.14, *SE* = 0.09, *p* = 0.108). Parental (*b* = 0.14, *SE* = 0.03, *p* < .001) and school norms (*b* = 0.18, *SE* = 0.03, *p* < .001) were to the same extent positively related to prosocial behaviour in both subgroups. However, peer norms had a significant positive effect on prosocial behaviour among minority participants (*b* = 0.16, *SE* = 0.04, *p* < .001) but not among majority participants (*b* = 0.06, *SE* = 0.04, *p* < .083) Interaction effect of peer norms and group status on prosocial behaviour in shown graphically in Fig 3.

**Fig 4:**
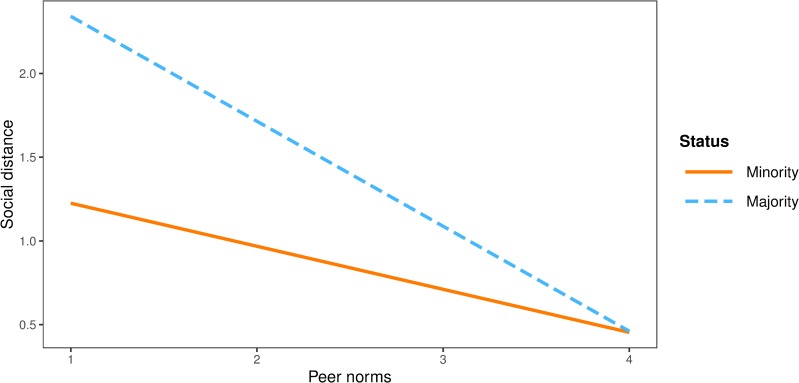
Interaction plot between peer norms and group status on social distance.

Finally, peer norms had a stronger negative effect on social distance among majority (*b* = -0.55, *SE* = 0.11, *p* < .001) than among minority participants (*b* = -0.29, *SE* = 0.08, *p* < .001). Interaction effect of peer norms and group status on social distance in shown graphically in Fig 4.

**Fig 5:**
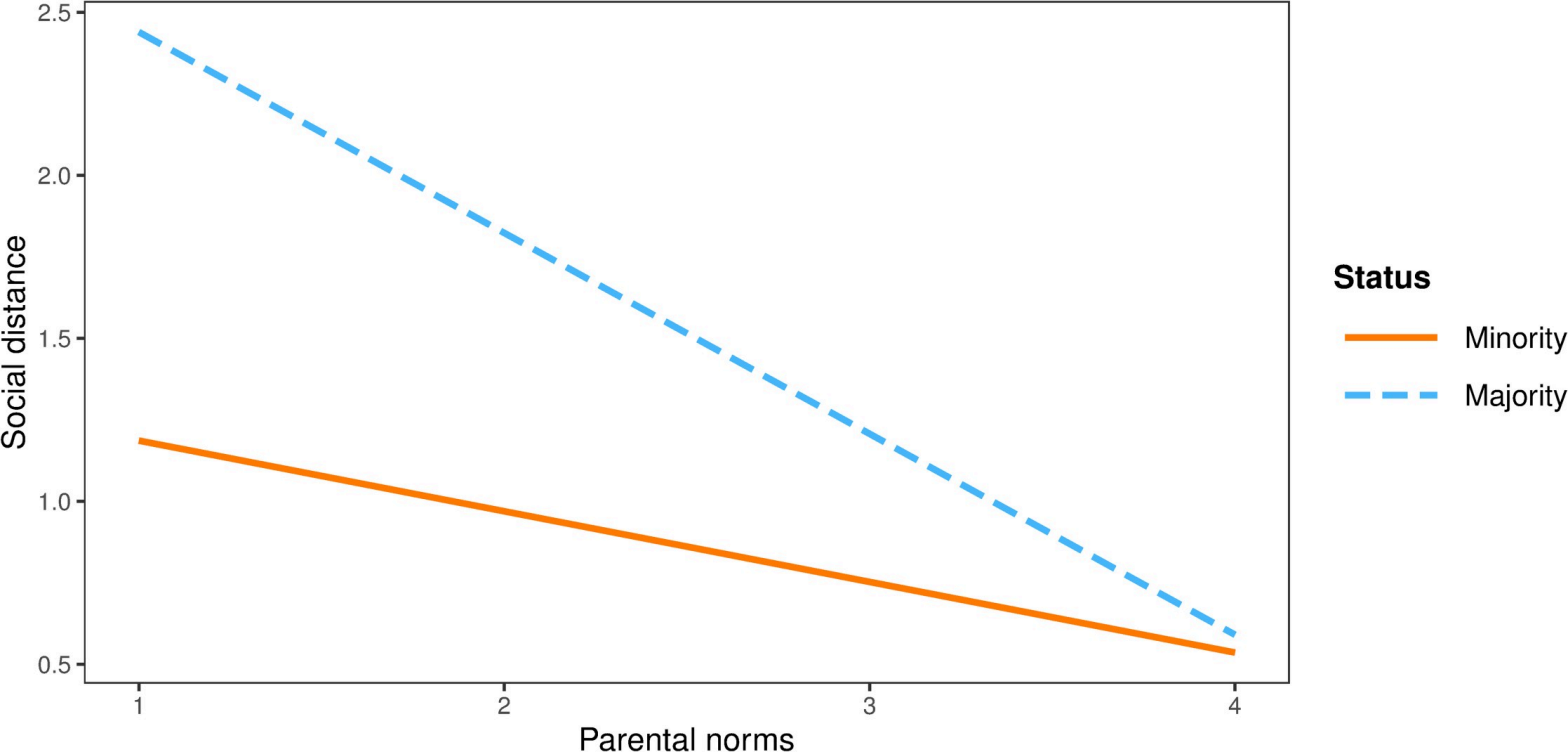
Interaction plot between parental norms and group status on social distance.

Accordingly, parental norms had stronger negative effect among majority (*b* = -0.59, *SE* = 0.10, *p* < .001) than among minority participants (*b* = -0.24, *SE* = 0.08, *p* = .002). Interaction effect of parental norms and group status on social distance in shown in Fig 5.

**Fig 6:**
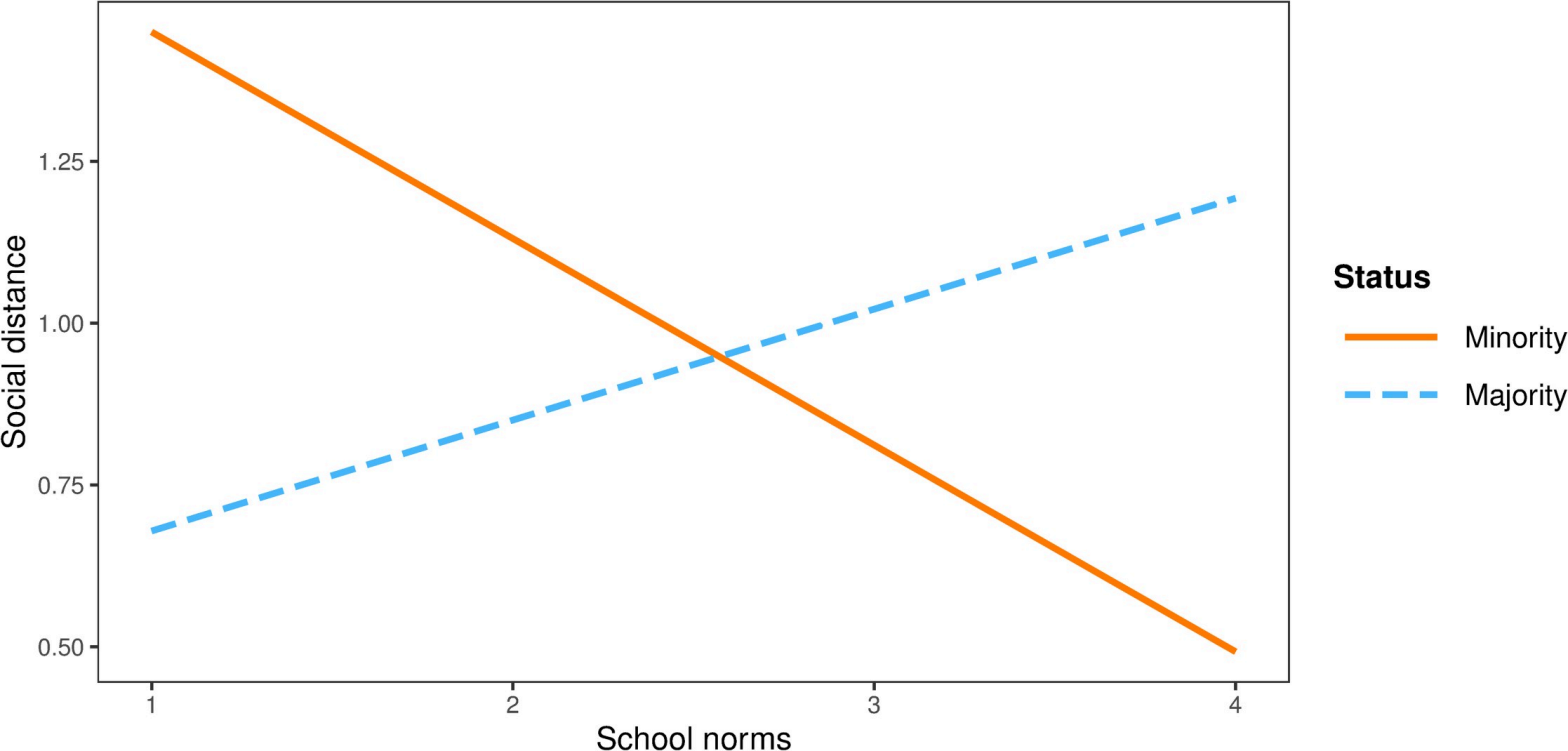
Interaction plot between school norms and group status on social distance.

However, the negative effect of school norms on social distance was significant for minority (*b* = -0.30, *SE* = 0.09, *p* = .001) but not for majority participants (*b* = 0.10, *SE* = 0.12, *p* = .399). Interaction effect of school norms and group status on social distance in shown graphically in Fig 6.

The partially constrained model for the majority participants explained 15% of variance in-group bias, 12% in prosocial behaviour, 9% in discrimination tendencies and 18% in social distance. The proportion of variance explained in all criterion variables, except for prosocial behaviour, was smaller for minority participants: 8% in in-group bias, 16% in prosocial behaviour, 5% in discrimination tendencies and 13% in social distance. The results suggest that group status moderates the effects of specific types of norms on specific types of intergroup behaviours. For minority groups, peer norms have a significant effect in predicting positive, prosocial behaviour and school norms in predicting negative, social distance outcome. Conversely, peer and parental norms have stronger effects in predicting social distance among the majority.

#### Moderation effects of intergroup context

The final multiple-group path analysis tested differences in strength of normative effects between four intergroup contexts in a similar way as the previous analysis for status differences. The constrained version of the model in which normative path coefficients were set to be equal among participants from the Croatian-Serbian, Croatian-Hungarian, Croatian-Czech and Croatian-Italian context had significantly worse fit than the unconstrained model (Δχ^2^(36) = 73.754, *p* < .001), indicating that intergroup context has an overall moderation effect. Follow-up tests of group differences in each regression path revealed that only path from parental norms to discrimination tendencies and path from parental norms to social distance differ significantly between intergroup contexts. Specifically, regression coefficients of these paths were different only between the subgroup of participants from the Croatian-Serbian context and those from all other intergroup contexts. The final partially constrained model in which only two normative paths were allowed to vary across two groups (participants from the Croatian-Serbian context and all other contexts) provided good overall fit and did not have a significantly different fit compared with unconstrained model (Δχ^2^(34) = 38.228, *p* = .283). Fit indices for the unconstrained, partially constrained and constrained model are reported in Table 5.

**Table 5:** Summary of fit statistics for the hypothesised multiple-group path model for intergroup context.

Model	S-B χ^2^	*df*	*p*	CFI	RMSEA	SRMR
Unconstrained	-	-	-	-	-	-
Partially constrained	38.228	34	.283	.99	.02	.04
Constrained	73.754	36	.000	.96	.07	.07

**Fig 7:**
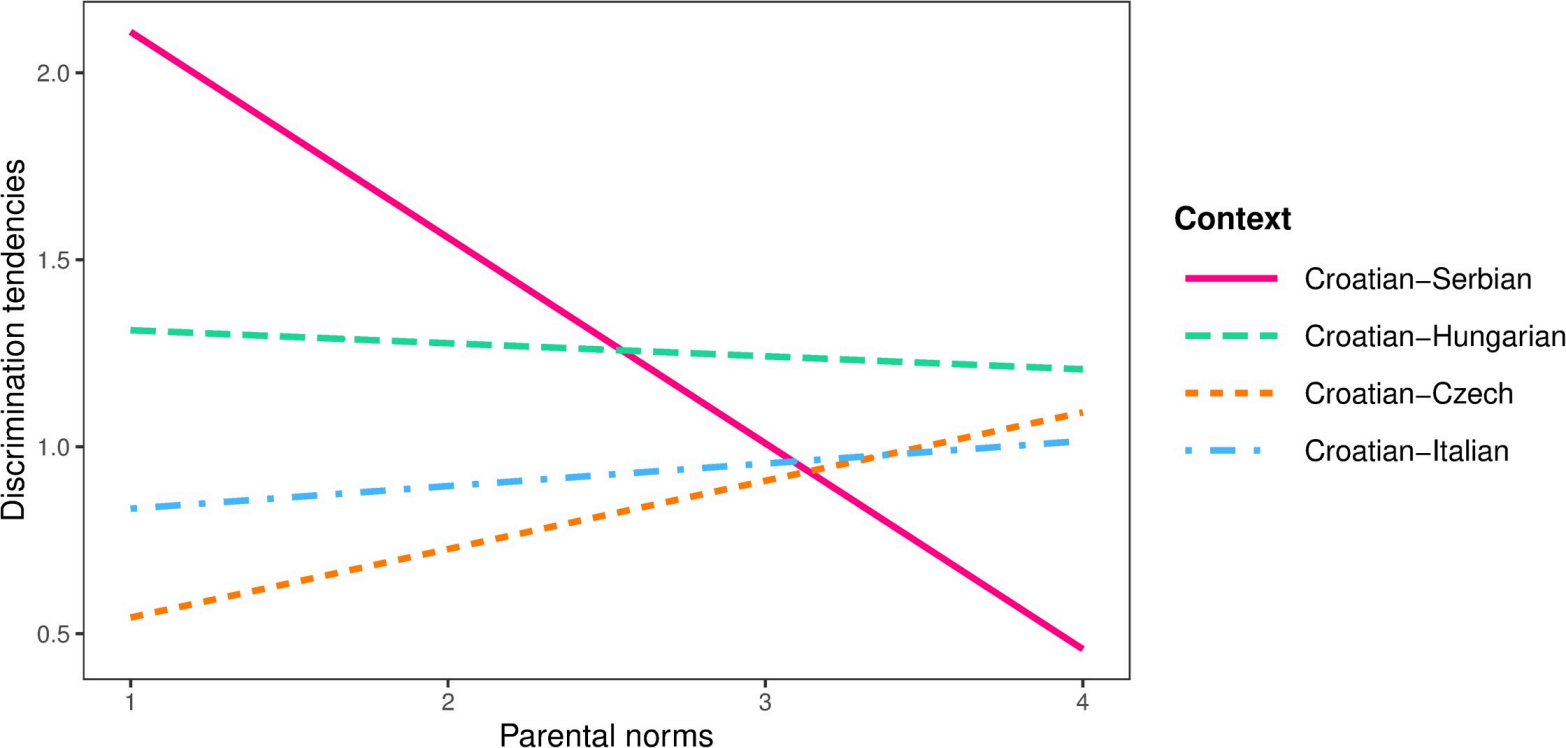
Interaction plot between parental norms and intergroup context on discrimination tendencies.

In the final model, peer (*b* = -0.39, *SE* = 0.12, *p* = .001) and parental norms (*b* = -0.78, *SE* = 0.10, *p* < .001) had equally strong negative effects on in-group bias in all intergroup contexts, while the effect of school norms was not significant (*b* = 0.07, *SE* = 0.16, *p* = .660). Although peer (*b* = -0.23, *SE* = 0.08, *p* = .004) and school norms (*b* = -0.23, *SE* = 0.09, *p* = .011) had equal negative effects on discrimination tendencies in all contexts, the effect of parental norms was significant among participants from the Croatian-Serbian context (*b* = -0.54, *SE* = 0.10, *p* < .001) and non-significant in all other intergroup contexts (*b* = 0.13, *SE* = 0.08, *p* = .090) Interaction effect of parental norms and intergroup context on discrimination tendencies in shown in Fig 7.

**Fig 8:**
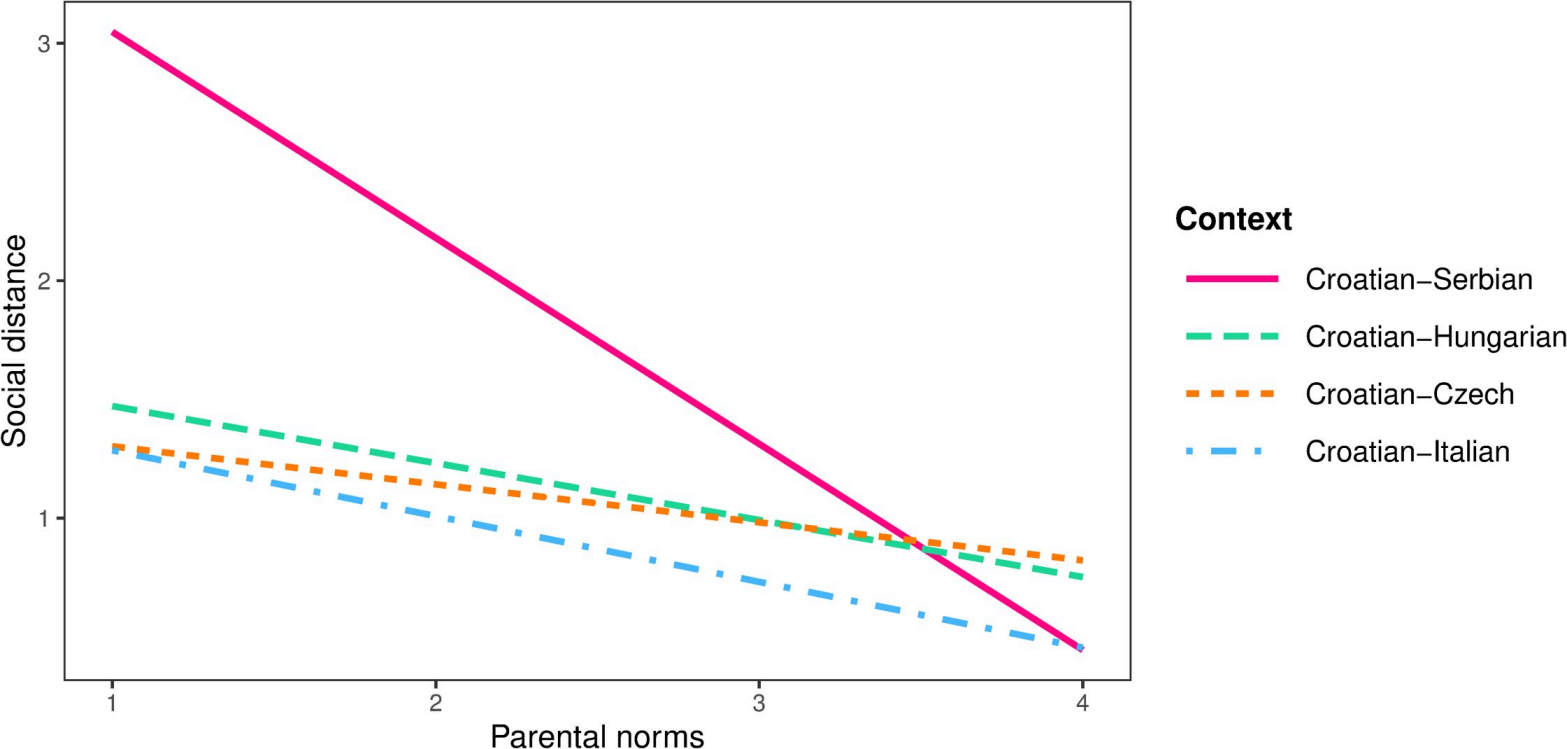
Interaction plot between parental norms and intergroup context on social distance.

Peer (*b* = 0.07, *SE* = 0.03, *p* = .011), parental (*b* = 0.10, *SE* = 0.03, *p* < .001) and school norms (*b* = 0.16, *SE* = 0.30, *p* < .001) were equally and positively related to prosocial behaviour in all contexts. The negative effects of peer norms (*b* = -0.43, *SE* = 0.08, *p* < .001) on social distance did not differ between the contexts, and school norms were not related to social distance in any of the contexts (*b* = -0.00, *SE* = 0.08, *p* = .998). However, parental norms had stronger negative effects on social distance for participants from the Croatian-Serbian context (*b* = -0.78, *SE* = 0.10, *p* < .001) than those from all other intergroup contexts (*b* = -0.26, *SE* = 0.09, *p* = .005). Interaction effects of parental norms and intergroup context on social distance in shown in Fig 8.

The proportion of variance explained for in-group bias, prosocial behaviour, discrimination tendencies and social distance was 9%, 8%, 14% and 23% for the subsample of participants from the Croatian-Serbian context; 11%, 12%, 2% and 10% for those from the Croatian-Hungarian context; 11%, 6%, 2% and 10% for the Croatian-Czech context, and 10%, 7%, 2% and 10% for the Croatian-Italian context respectively. The hypothesis predicting stronger normative effects on intergroup outcomes in post-conflict context was partially supported given that the significant difference between the Croatian-Serbian context and all other intergroup contexts were found only in the relation between parental norms about intergroup contact and two different negative intergroup outcomes (discrimination tendencies and social distance).

## Discussion

The purpose of this research was to examine the relative effects of peer, parental and school norms about intergroup contact on positive and negative indicators of intergroup relations in a diverse sample of majority and minority adolescents of various ages living in four different majority-minority contexts. In an attempt to extend previous findings and determine boundary conditions under which normative effects are attenuated, we also tested for potential age, social status and contextual differences in the strength of normative effects.

We predicted that positive perception of all three types of norms will be negatively related to in-group bias, social distance and tendency to discriminate the outgroup, and positively related to outgroup prosocial behaviour. In support of our hypothesis, all three types of norms significantly predicted lower levels of discrimination tendencies and higher levels of prosocial behaviour towards the outgroup among the total sample. Moreover, all three types of norms had approximately equal effects on these dependent variables. However, only peer and parental norms predicted lower in-group bias and social distance towards the outgroup. These results are in line with previous research showing that peer groups [16], family members [15], and school environments [36], when examined separately, all play important roles in adolescents’ interethnic relations. However, our results extend previous findings by demonstrating independent effects of different types of norms in the prediction of intergroup attitudes and behaviours. Moreover, the fact that we found significant normative effects in the sample of majority and minority adolescents attending separate schools or separate classes within the same school emphasises the importance of positive contact norms in contexts which limit the opportunity for direct intergroup contact. This seems to suggest that mere perception that one’s peers, parents and school authorities have or approve intergroup contact can benefit adolescents’ intergroup relations even if they themselves have reduced possibilities of experiencing meaningful contact with outgroup members within the school.

Our findings are also consistent with studies indicating differential effects of peer and school norms. For example, Tropp et al. [9] and McKeown and Taylor [31] found peer norms to be a more consistent predictor of adolescents’ contact experiences and intergroup behaviours than school norms. In our study, school norms did not predict attitudinal measures of bias towards one’s own group and distance from the outgroup. However, they did predict outcomes that show more behavioural tendencies, both positive (prosocial behaviour) and negative (tendency toward discrimination). Although there isn’t much research examining the differential impact of school norms on attitudinal and behavioural intergroup outcomes, it might be that schools, through implementation of regulations and rules regarding appropriate and undesirable conduct, might be more influential on students’ behavioural intentions and actual (or at least self-presentational) behaviour than of less observable attitudinal outcomes. If this is true, school norms, as the type of norm most amenable to change by external factors, could be used to prevent negative and promote desirable intergroup behaviours, even when minority and majority students are physically separated within their school environment. For example, in schools with different majority and minority classes, school staff could send direct and indirect messages about the desirability of intergroup contact by modelling positive intergroup interactions, organising shared intergroup activities, as well as by sanctioning intergroup conflicts. Similarly, exclusive majority and minority schools could encourage mutual cooperation through participation in joint projects and activities within the wider local community. However, one may wonder if the effect of school norms about intergroup contact would be equally robust if measures of prosocial and discriminatory behaviours contained more items describing situations outside the school context. Hence, further research could include measures that will enable to test if school norms about contact are behavioural guidance for school behaviours only or they also, once adopted, efficiently guide interethnic behaviour of adolescents in the out-of-school contexts.

Regarding the role of potential moderator variables, we expected stronger effects of perceived peer norms among younger adolescents given that resistance to peer influence increases linearly throughout adolescence [33,34]. In addition, we did not expect age to moderate the effect of parental norms. In a partial support of our hypothesis, age moderated the effects of peer norms in the presumed direction only on social distance but did not have an effect on the relation between peer norms and any other dependent variable. More research is thus needed to examine why all intergroup outcomes do not follow the same age trend related to the effects of peer norms. As expected, parental norms had stable effects on all adolescents’ intergroup outcomes regardless of their age. Although we did not examine age-related changes in the effects of peer and parental norms, our comparison of normative effects among adolescents of different ages supports longitudinal findings indicating that parents influence their children’s attitudes throughout their adolescence, while peers have more impact on early adolescents [35].

This research has also explored whether the patterns of normative effects are similar or different for ethnic majority and minority adolescents, given prior mixed results on the role of group status as a potential moderator of these effects. Group status was a significant moderator in the relation between peer norms and prosocial behaviour, as well as in the relation between all three types of norms and social distance. Specifically, positive peer norms significantly predicted prosocial behaviour, and positive school norms predicted lower social distance only among ethnic minority adolescents. By contrast, positive peer and parental norms more strongly predicted lower social distance among majority, than among minority adolescents. Normative effects on other intergroup outcomes did not depend on group status. Our finding of stronger peer normative effects on social distance in the sample of majority adolescents complements previous studies indicating that perceived peer norms supporting intergroup contact are more strongly related to preferences for cross-ethnic friendships and comfort with out-group members among ethnic majority students [9,39]. Moreover, our study shows that not only peer, but also parental norms have more impact on the majorities’ acceptance of close relationships with members of the minority than vice versa. However, the effect of school norms on social distance in our study was significant only for minority students. These results are in line with previous research corroborating differences in the importance of peer and school norms for ethnic majority and minority groups. For example, Tropp et al. [9] found that peer norms more strongly predict comfort with the outgroup among majority adolescents, while school norms more strongly predict the number of cross-ethnic friends among the minority. Thus, it seems that majority adolescents, when deciding whether to have close relationships with the outgroup, rely more on proximal sources of normative influence, such as peers and parents, while minority adolescents depend more on norms they perceive in their broader, school environment. It may be, as Tropp et al. [9] have suggested, that formal school authorities are more influential on minority students’ intergroup relations due to their lower social status and generally less positive intergroup experiences.

To our knowledge, this is the first research showing majority-minority differences in peer influences on positive, prosocial behaviours. Although more research is needed to explain these findings, it may be that minority adolescents’ awareness of their lower social status makes them more sensitive to environmental messages concerning behaviours which could improve their relations with the majority. For instance, Guinote, Cotzia, Sandhu and Siwa [59] have shown that low status individuals, compared to their higher status counterparts, exhibit more prosocial behaviour and associated goals and values. The authors interpreted this tendency as a way to increase their social status and to regulate social interactions with individuals of higher status. If intergroup prosocial behaviour is valued more among minority groups, it should also be strongly regulated by in-group norms supporting positive relations with the outgroup. Given that our measure of prosocial behaviour captures prosocial tendencies in the context of peer interactions, it is therefore not surprising that we found status differences in the effects of only peer norms. However, it would be interesting to further investigate if different types of in-group norms regarding contact with the outgroup have significant effects on a wider range of positive intergroup behaviours among minority adolescents.

Finally, this is the first research examining potential contextual moderation of normative effects in a single study with the assumption that norms will generally have stronger effects in intergroup contexts with history of a recent intergroup conflict. In a support of our hypothesis, we found that parental norms were associated with lower discrimination tendencies only among adolescents living in ethnically divided, post-conflict communities in the Croatian-Serbian context. Additionally, parental normative influences on social distance were stronger in this post-conflict context compared to all other, relatively harmonious intergroup contexts. These findings offer support for the view that in-group norms are likely to be most influential when in-group identification is increased as a result of intergroup conflict [60]. Moreover, our findings seem to suggest that adolescents living in such contexts may be especially attuned to parental normative influences, when deciding whether to act negatively (i. e. to discriminate or distance themselves from the outgroup) towards members of the outgroup. Nonetheless, we did not take degree of ethnic identification into account as an antecedent of stronger normative effects in post-conflict communities, so further research is needed to specify the factors contributing to the heightened effects of specific types of norms in such environments.

Taken together, our findings demonstrate that although all three types of norms can play an important role in adolescents’ intergroup attitudes and behaviours, their relative importance seems to vary depending on the specific type of intergroup outcome, social status of the groups involved, the type of intergroup contexts in which these norms operate, and sometimes even the age of the normative target. However, there are some limitations to this study that need to be acknowledged.

First, while the overall sample size, as well as the size of status and contextual subsamples was quite large, we did not have enough minority and majority adolescents from each intergroup context to differentiate between them in multi-group analyses. Therefore, we cannot claim that majority-minority differences in the strength of normative influences obtained on aggregate samples of minority and majority adolescents would be replicated in each intergroup context. In addition, it is possible that the comparison of majorities (or minorities) from different multi-ethnic communities would reveal additional contextual differences that were concealed by contextually comparing all adolescents, regardless of their status.

Second, the relations between different types of norms and intergroup outcomes were only examined cross-sectionally, which limits our understanding of causal, as well as long-term effects of norms about intergroup contact. Longitudinal research is thus needed to test whether the same normative effects would be obtained over time. In addition, longitudinal research including more than two time points could provide more in-depth insight into age-related changes in the effects of different types of norms. Nevertheless, cross-sectional research can be useful in order to determine boundary conditions where established relationships between the variables of interest are attenuated [61], which is exactly what we have done through the use of moderator tests.

The third limitation concerns the relatively low and variable internal reliabilities of peer and school norms subscales across different status and contextual subsamples which could have impaired their predictive validity. However, this may suggest that the effects of normative variables in the current study might be even stronger than reported. Future research should therefore modify the current measure of normative influences, by increasing the number of items on each subscale.

Finally, peer, parental and school norms about intergroup contact have been assessed using explicit reports of participant’s perceptions of norms which some authors claim to be susceptible to various individual biases [62]. Future research could benefit from including more implicit measures of normative influences, such as measures of peer, parental or teacher attitudes towards intergroup interactions or even self-reported number of intergroup friendships. However, some authors have argued that individuals’ perception of norms within their community may not match the actual attitudes or behaviours of community members, and that these subjective perceptions may have even more influence on individual’s subsequent attitudes and behaviours [63].

Despite the limitations, we believe that this research contributes to the existing literature on contact related normative influences, by demonstrating how different types of norms predict intergroup outcomes of ethnic minority and majority youth and by establishing the conditions under which such norms may be more or less influential on adolescents’ intergroup attitudes and behaviours.

## Supporting information

S1 Dataset Dataset used to obtain the research results. (XLSX)

S1 Questionnaire The croatian and english language version of the questionnaire. (PDF)
