# Correction: The role of peer, parental, and school norms in predicting adolescents’ attitudes and behaviours of majority and different minority ethnic groups in Croatia

**DOI:** 10.1371/journal.pone.0228970

**Published:** 2020-02-04

**Authors:** Lana Pehar, Dinka Čorkalo Biruški, Tea Pavin Ivanec

The ORCID iDs are missing for the second and third author. Please see the authors’ respective ORCID iDs here:

- Author Dinka Čorkalo Biruški’s ORCID iD is: 0000-0002-2954-4349 (https://orcid.org/0000-0002-2954-4349).
- Author Tea Pavin Ivanec’s ORCID iD is: 0000-0003-3225-2272 (https://orcid.org/0000-0003-3225-2272).
