# Supplementary material for: The role of peer, parental, and school norms in predicting adolescents’ attitudes and behaviours of majority and different minority ethnic groups in Croatia
Source: PLoS One. 2020 Jan 10;15(1):e0227512. doi: 10.1371/journal.pone.0227512 (PMC6953838; doi:10.1371/journal.pone.0227512)
Supplement: S1 Questionnaire — (PDF) [file pone.0227512.s002.pdf]

Supplementary Material

I. The Croatian language version of the questionnaire

Zahvaljujemo ti što si pristao/la sudjelovati u ovom istraživanju koje ispituje kako se osjećaš u školi, među svojim vršnjacima i kakvi su vaši odnosi. U ovom upitniku nalazi se niz pitanja grupiranih u cjeline, a ispred svake cjeline je posebna uputa. U nekim dijelovima upitnika trebat ćeš procijeniti koliko se slažeš ili ne slažeš s pojedinim tvrdnjama. U drugim ćeš pitanjima trebati odabrati onaj odgovor koji se najviše odnosi na tebe. Molimo te da svako pitanje pažljivo pročitaš, odabereš samo jedan od ponuđenih odgovora te da odgovaraš iskreno. Tvoji su odgovori potpuno povjerljivi i vidjet će ih samo istraživači – nitko više.

Socijalna distanca

Sada te molimo da iskažeš na kakve bi odnose bio/bila spreman/a s pripadnicima različitih naroda. Imena tih naroda napisana su u lijevom stupcu u donjoj tablici. Vrste odnosa koje ljudi mogu međusobno imati napisane su u najgornjem retku tablice. **Molimo te da za svakog pripadnika naroda s lijeve strane zaokružiš „da“ ili „ne“ za svaki odnos označen u najgornjem retku tablice, ovisno o tome bi li na takav odnos bio/bila spreman/a ili ne.**

Npr. ako bi s pripadnikom nekog naroda bio/bila spreman/a i živjeti u istoj državi i ići u isti razred i stanovati kuća do kuće i tako dalje, tada ćeš u svim kućicama zaokružiti DA. Ako, s druge strane, s pripadnikom nekog naroda ne bi bio/bila spreman/a ni na kakve odnose, tada ćeš u svim kućicama zaokružiti NE.

Moguće su, naravno, i druge kombinacije, važno je samo da **za svaki odnos *na koji bi bio/bila spreman/a zaokružiš DA*, a za svaki odnos na koji *ne bi bio/bila spreman/a zaokružiš NE.***

	Da živimo u Hrvatskoj		Da idemo u isti razred		Da stanujemo kuća do kuće		Da smo prijatelji i da se družimo izvan kuće		Da smo prijatelji i da se posjećujemo		Da smo rođaci/ svojta		Da mi je cura/dečko	
Hrvati	da	ne	da	ne	da	ne	da	ne	da	ne	da	ne	da	ne
Srbi	da	ne	da	ne	da	ne	da	ne	da	ne	da	ne	da	ne
Mađari	da	ne	da	ne	da	ne	da	ne	da	ne	da	ne	da	ne
Česi	da	ne	da	ne	da	ne	da	ne	da	ne	da	ne	da	ne
Talijani	da	ne	da	ne	da	ne	da	ne	da	ne	da	ne	da	ne

Unutargrupne norme o međugrupnom kontaktu

M. U dolje navedenim tvrdnjama opisuju se neka ponašanja koja mogu pokazivati djeca i odrasli iz **tvoje nacionalne grupe**. Molimo te da procijeniš koliko se slažeš ili ne slažeš s dolje navedenim tvrdnjama. Uz svaku tvrdnju zaokruži jedan broj koji najbolje opisuje koliko se slažeš ili ne slažeš s tom tvrdnjom. Pri tome brojevi imaju sljedeće značenje:

1 – izrazito se ne slažem
2 – uglavnom se ne slažem
3 – uglavnom se slažem
4 – izrazito se slažem

1.	Većina mojih prijatelja izbjegava vršnjake hrvatske/srpske/mađarske/češke/ talijanske nacionalnosti.	1 2 3 4
2.	Moji prijatelji često idu van s vršnjacima hrvatske/srpske/mađarske/češke/talijanske nacionalnosti.	1 2 3 4
3.	Ljubavne veze između mojih vršnjaka Hrvata i Srba/Mađara/Čeha/Talijana su uobičajene.	1 2 3 4
4.	Moji vršnjaci smatraju da ne treba imati bliske prijatelje hrvatske/srpske/mađarske/ češke/talijanske nacionalnosti.	1 2 3 4
5.	Mojim prijateljima bi smetalo da u naše društvo dovedem osobu hrvatske/srpske/ mađarske/češke/talijanske nacionalnosti.	1 2 3 4
6.	Moji roditelji se druže s pripadnicima hrvatske/srpske/mađarske/češke/talijanske nacionalnosti.	1 2 3 4
7.	Moji roditelji imaju bliske prijatelje hrvatske/srpske/mađarske/češke/talijanske nacionalnosti.	1 2 3 4
8.	Moji roditelji podržavaju da se družim sa svima, bez obzira na nacionalnost.	1 2 3 4
9.	Mojim roditeljima ne bi bilo drago da u našu kuću pozivam vršnjake hrvatske/ srpske/mađarske/češke/talijanske nacionalnosti.	1 2 3 4
10.	Moji roditelji ne bi odobrili da moj/a cura/dečko bude hrvatske/srpske/mađarske/ češke/talijanske nacionalnosti.	1 2 3 4
11.	U mojoj školi je uobičajeno da se učenici hrvatske i srpske/mađarske/češke/ talijanske nacionalnosti druže..	1 2 3 4
12.	Nastavnici hrvatske i srpske/mađarske/češke/talijanske nacionalnosti u mojoj školi dobro surađuju.	1 2 3 4
13.	U mojoj školi se u različite aktivnosti uvijek nastoji uključiti sve učenike, bez obzira na njihovu nacionalnost.	1 2 3 4
14.	Naši nastavnici u školi potiču nas da se družimo s vršnjacima hrvatske/srpske/ mađarske/češke/talijanske nacionalnosti.	1 2 3 4
15.	Naš/a ravnatelj/ica potiče suradnju među učenicima i nastavnicima hrvatske i srpske/mađarske/češke/talijanske nacionalnosti.	1 2 3 4

Unutargrupna pristranost

Molimo te da iskažeš svoj opći odnos prema navedenim narodima koji žive u Hrvatskoj. Svoje procjene daj na skali od 0 do 10. Npr. ako tvoj opći odnos prema navedenom narodu nije ni negativan ni pozitivan, zaokruži broj 5.

1. Hrvati/Srbi/Mađari/Česi/Talijani u Hrvatskoj:

0 ------ 1 ------ 2 ------ 3 ------ 4 ------ 5------ 6 ------ 7------ 8 ------ 9 ------ 10

negativan | ni negativan ni pozitivan | pozitivan

2. Hrvati/Srbi/Mađari/Česi/Talijani u Hrvatskoj:

0 ------ 1 ------ 2 ------ 3 ------ 4 ------ 5------ 6 ------ 7------ 8 ------ 9 ------ 10

negativan | ni negativan ni pozitivan | pozitivan

Sklonost diskriminaciji vanjske grupe

Niže su u obliku tvrdnji opisane različite situacije u kojima se ljudi mogu naći. Molimo te da za svaku situaciju zamisliš da se događa tebi. Pročitaj i za svaku tvrdnju odgovori slažeš li se s njom ili ne, i to tako da zaokružiš DA ako se slažeš s tvrdnjom, odnosno ako bi tako postupio/la u opisanoj situaciji ili da zaokružiš NE, ako se ne slažeš s tvrdnjom, odnosno ako ne bi tako postupio/la u opisanoj situaciji.

1. Da mi nastavnica da zadatak da pomognem jednom od dvojice učenika koji nisu bili u školi tako da jednom od njih kući odnesem zadaću, ja bih odabrao/la odnijeti zadaću Hrvatu/Srbinu/Mađaru/Čehu/Talijanu, premda Hrvat/Srbin/Mađar/Čeh/Talijan stanuje bliže.

DA NE

2. Da ja trebam odlučiti koji će učenici biti izabrani u školsku sportsku momčad, ja bih, između dva učenika, izabrao/la Hrvata/Srbina/Mađara/Čeha/Talijana, premda je malo slabiji sportaš/ica.

DA NE

3. Da mi se sviđa neka djevojka/dečko kojeg ne poznajem, odlučio/la bih pristupiti joj/mu jedino ako je moje nacionalnosti.

DA NE

4. Na važnom natjecanju iz matematike radije bih radio/la u paru s Hrvatom/Srbinom/Mađarom/Čehom/Talijanom, premda znam da je Hrvat/Srbin/Mađar/Čeh/Talijan bolji matematičar/ka.

DA NE

5. Da sam zaboravio/la ponijeti nešto od pribora za školu, posudio/la bih to jedino od Hrvata/Srbina/Mađara/Čeha/Talijana.

DA NE

6. Da sam zaboravio/la napisati zadaću, radije bih dobio/la jedinicu, nego zadaću prepisao/la od Hrvata/Srbina/ Mađara/Čeha/Talijana.

DA NE

7. Na školskom izletu bih uvijek radije dijelio/la sobu s Hrvatom/Srbinom/Mađarom/Čehom/Talijanom iako znam da je Hrvat/Srbin/Mađar/Čeh/Talijan zabavniji/a.

DA NE

8. Na fejsu/društvenim mrežama bih prihvatio/la zahtjev za prijateljstvom samo ako dolazi od Hrvata/Srbina/Mađara/ Čeha/Talijana.

DA NE

<u>Prosocijalno ponašanje</u>

Sada te molimo da promisliš kako se ponašaš kada se nađeš u situacijama koje su opisane u lijevoj koloni donje tablice. Ako nikada nisi bio/la u takvoj situaciji, molimo te da zamisliš kako bi se u njoj ponašao/la. Uz svaku situaciju zaokruži jedan broj koji najbolje opisuje tvoju reakciju u toj situaciji:

		Podržim ih ili im se pridružim	Pravim se da ne primjećujem/ ignoriram ih	Tražim od njih da prestanu	Zamolim moje vršnjake da pomognu da to prestane
1.	Kada moji vršnjaci šire laži ili ogovaraju učenike hrvatske/ srpske/mađarske/češke/talijanske nacionalnosti.	1	2	3	4
2.	Kada moji vršnjaci vrijeđaju učenike hrvatske/srpske/ mađarske/češke/talijanske nacionalnosti.	1	2	3	4
3.	Kada moji vršnjaci provociraju učenike hrvatske/srpske/ mađarske/češke/talijanske nacionalnosti isticanjem nacionalnih simbola.	1	2	3	4
4.	Kada moji vršnjaci prijete učenicima hrvatske/srpske/ mađarske/češke/talijanske nacionalnosti.	1	2	3	4
5.	Kada se moji vršnjaci potuku s učenicima hrvatske/srpske/ mađarske/češke/talijanske nacionalnosti.	1	2	3	4

II. The English language version of the questionnaire

Thank you for agreeing to participate in this research that examines how you feel at school, among your peers, and how are your relationships. This questionnaire contains a series of questions grouped into units, with specific instructions in front of each unit. In some parts of the questionnaire, you will need to evaluate how much you agree or disagree with each statement. In other questions you will need to choose the one that is most relevant to you. Please read each question carefully, select only one of the answers offered, and answer honestly. Your answers are completely confidential and will only be seen by researchers - no one else.

Social distance towards the outgroup

We now ask you to indicate what kind of relationships are you willing to have with members of different nations. The names of these nations are written in the left column in the table below. The types of relationships that people can have with each other are written in the top row of the table. **For each member of nations on the left and for each relationship indicated in the top row of the table, please circle "YES" or "NO", depending on whether or not you would be willing to have such a relationship.**

Eg. if you would be willing to live in the same state with a member of a specific nation and go to the same class and live as a next-door neighbour, and so on, then you would circle YES in all the boxes. If, on the other hand, you would not be willing to have any of the relationships with a member of a specific nation, then you would circle NO in all the boxes.

Of course, other combinations are also possible, **it is only important that for each relationship you are willing to have to circle YES, and for each relationship you are not willing to have to circle NO.**

	To live together in Croatia		To go to the same class		To live as a next-door neighbour		To be friends and socialise outside your home		To be friends who visit each other at home		To be relatives		To have him/her as a boyfriend/ girlfriend	
Croats	yes	no	yes	no	yes	no	yes	no	yes	no	yes	no	yes	no
Serbs	yes	no	yes	no	yes	no	yes	no	yes	no	yes	no	yes	no
Hungarians	yes	no	yes	no	yes	no	yes	no	yes	no	yes	no	yes	no
Czechs	yes	no	yes	no	yes	no	yes	no	yes	no	yes	no	yes	no
Italians	yes	no	yes	no	yes	no	yes	no	yes	no	yes	no	yes	no

In-group norms about intergroup contact

The statements below describe some behaviours that children and adults **in your national group** can show. Please evaluate how much you agree or disagree with the statements below. Circle one number next to each statement which best describes how much you agree or disagree with that statement. The numbers have the following meaning:

1 – I completely disagree
2 – I mostly disagree
3 – I mostly agree
4 – I completely agree

1.	Most of my friends avoids peers of Croatian/Serbian/Hungarian/Czech/Italian nationality.	1 2 3 4
2.	My friends usually go out with peers of Croatian and Serbian/Hungarian/Czech/ Italian nationality.	1 2 3 4
3.	Romantic relationships between my peers of Croatian and Serbian/Hungarian/ Czech/Italian nationality are common.	1 2 3 4
4.	My peers think that one shouldn't have close friends of Croatian/Serbian/ Hungarian/Czech/Italian nationality.	1 2 3 4
5.	My friends would mind if I invited a peer of Croatian/Serbian/Hungarian/Czech/ Italian nationality into our circle.	1 2 3 4
6.	My parents hang out with individuals of Croatian/Serbian/Hungarian/Czech/Italian nationality.	1 2 3 4
7.	My parents have close friends of Croatian/Serbian/Hungarian/Czech/Italian nationality.	1 2 3 4
8.	My parents support me in hanging out with everyone, regardless of their nationality.	1 2 3 4
9.	My parents would not be pleased if I invited peers of Croatian/Serbian/Hungarian/ Czech/Italian nationality to our house.	1 2 3 4
10.	My parents would not approve if my girlfriend/boyfriend was of Croatian/Serbian/ Hungarian/Czech/Italian nationality.	1 2 3 4
11.	In my school it is common that students of Croatian and Serbian/Hungarian/Czech/ Italian nationality hang out with each other.	1 2 3 4
12.	Teachers of Croatian and Serbian/Hungarian/Czech/Italian nationality cooperate well at my school.	1 2 3 4
13.	In my school all students are included in different activities, regardless of their nationality.	1 2 3 4
14.	Our teachers encourage us to socialise with our peers of Croatian/Serbian/ Hungarian/Czech/Italian nationality.	1 2 3 4
15.	Our principal encourages cooperation between students and teachers of Croatian and Serbian/Hungarian/Czech/Italian nationality.	1 2 3 4

In-group bias

We ask you to state your general attitude towards these nations living in Croatia. Give your estimates on a scale from 0 to 10. Eg. if your general attitude towards the stated nation is neither negative nor positive, circle number 5.

1. Croats/Serbs/Hungarians/Czechs/Italians in Croatia:

0 ------ 1 ------ 2 ------ 3 ------ 4 ------ 5------ 6 ------ 7------ 8 ------ 9 ------ 10

negative — neither negative, nor positive — positive

2. Croats/Serbs/Hungarians/Czechs/Italians in Croatia:

0 ------ 1 ------ 2 ------ 3 ------ 4 ------ 5------ 6 ------ 7------ 8 ------ 9 ------ 10

negative — neither negative, nor positive — positive

Tendency to discriminate against the outgroup

The statements below describe the various situations in which people can find themselves. Please, for every situation, imagine that it is happening to you. Read and for each statement answer whether you agree with it or not, by circling YES if you agree with the statement, that is, if you would do so in the situation described above, or by circling NO if you disagree with the statement, or if would not do so in the described situation.

1. If the teacher asked me to help one of the two students who were absent from the school by bringing the homework to one of them, I would choose to bring the homework to Croat/Serb/Hungarian/Czech/Italian although the Croat/Serb/Hungarian/Czech/Italian lives closer to me.

YES NO

2. If I were to decide which students would be selected on the school sports team, between the two students I would choose the Croat/Serb/Hungarian/Czech/Italian student, although he is a slightly weaker athlete.

YES NO

3. If I liked a girl/boy I did not know, I would only choose to approach her/him if she/he is of my nationality.

YES NO

4. On an important math competition, I would rather work in pair with a Croat/Serb/Hungarian/Czech/Italian, although I know that the Croat/Serb/Hungarian/Czech/Italian is a better mathematician.

YES NO

5. If I forgot to bring some of my school supplies, I would borrow it only from a Croat/Serb/Hungarian/Czech/Italian.

YES NO

6. If I forgot to write my homework, I would rather get an F than copy it from a Croat/Serb/Hungarian/Czech/Italian.

YES NO

7. On a school trip, I would always prefer to share a room with a Croat/Serb/Hungarian/Czech/Italian, even though I know that the Croat/Serb/Hungarian/Czech/Italian is more fun.

YES NO

8. On Facebook/social networks, I would only accept a friend request if it came from a Croat/Serb/Hungarian/Czech/ Italian.

YES NO

Prosocial behaviour towards the outgroup

Now we ask you to consider how you act when you find yourself in situations described in the left column of the table below. If you have never been in such a situation, please imagine how you would behave in it. Next to each situation, circle one number that best describes your reaction in that situation

		I support them or join them	I pretend not to notice/I ignore them	I ask them to stop	I ask my peers to help me stop it
1.	When my peers spread lies about or gossip students of Croatian/Serbian/ Hungarian/Czech/Italian nationality.	1	2	3	4
2.	When my peers insult students of Croatian/Serbian/Hungarian/Czech/Italian nationality.	1	2	3	4
3.	When my peers provoke students of Croatian/Serbian/Hungarian/Czech/Italian nationality by emphasizing national symbols.	1	2	3	4
4.	When my peers threaten students of Croatian/Serbian/Hungarian/Czech/Italian nationality.	1	2	3	4
5.	When my peers get into a fight with students of Croatian/Serbian/Hungarian/ Czech/Italian nationality.	1	2	3	4

!
