# Supplementary material for: The role of peer, parental, and school norms in predicting adolescents’ attitudes and behaviours of majority and different minority ethnic groups in Croatia
Source: PLoS One. 2020 Jan 10;15(1):e0227512. doi: 10.1371/journal.pone.0227512 (PMC6953838; doi:10.1371/journal.pone.0227512)
Supplement: Attachment — Submitted filename: Response to Reviewer.docx [file pone.0227512.s003.docx]

Lana Pehar
Department of Psychology
Faculty of Humanities and Social Sciences
University of Zagreb

Zagreb, Croatia

lpehar@ffzg.hr

Daniel Romer
Academic Editor
PLOS ONE

December 19, 2019.

**Subject: Revision and resubmission of manuscript PONE-D-19-16051_R1**

Dear Dr. Romer,

thank you for accepting our first revision of the manuscript „Normative approach to intergroup relations: The role of peer, parental and school norms about intergroup contact in predicting adolescents’ interethnic attitudes and behaviours “, as well as for your additional insights on how to make our manuscript stronger and clearer. We have made appropriate changes to the manuscript after the second review which reflect your helpful suggestions. The changes were highlighted in the revised manuscript using track changes, as you requested.

Here is a point-by-point response to your comments and concerns.

**Comment 1:** I think your paper will have relevance to educators and others interested in trying to improve interethnic relations in countries such as Croatia. So, I wonder if you might consider a title that makes it clearer that you are studying these issues in a country that has had significant interethnic conflict, something like: The role of peer, parental, and school norms in predicting adolescents’ attitudes and behaviours for both majority and different minority ethnic groups in Croatia. I of course leave this up to you, but I think it will make the abstract a bit more interesting as well.

**Response:** Thank you for this excellent observation, we have changed the title of our manuscript according to your proposal on first page of the manuscript (lines 4 to 5 in the revised manuscript).

**Comment 2:** I also think that the types of schools you are studying will be of interest because the minority youth are segregated from the majority youth in their classes. This would seem to reduce intergroup contact and thus make it more difficult to reduce barriers between the groups. You can’t tell if that matters, but I think it is something that might be worth noting in the discussion.

**Response:** We agree with this and have incorporated a comment relating to specific school contexts in our study in the discussion section of the revised manuscript (lines 790 to 797).

**Comment 3:** Related to this point, you seem to downplay the positive role of school norms in your results. On page 35 lines 772-773, you suggest that the significant effects of school norms are due to “a common method factor.” This is confusing because if anything the common variance shared by school norms with the other norms should make it more difficult to detect any effects of school norms. So, the fact that you got relations beyond peer and parental norms for behavioural outcomes is worth highlighting rather than downplaying. Also, this is a factor that is most amenable to intervention, and so it would be worth highlighting it in the introduction as well as the discussion. That is, is there a role for schools despite the segregation of students and the history of conflict to encourage greater intergroup acceptance?

**Response:** We appreciate your concern regarding our explanation of the school norms effect. However, we did not want to say that the significant effect of the school norms is due to a common method factor shared by different measures of contacts norms, but instead due to common variance shared by our measure of school norms and measures of discrimination tendencies and prosocial behaviour (all three measures include items related to the school context). We rephrased this part in our revised manuscript to make it clearer, as well as to avoid the “common method factor” implications (lines 818 to 823). In addition, we agree with your observation that we did not sufficiently emphasize the positive role of school norms in our results. Accordingly, we highlighted the potential of school norms in improving intergroup relations, both in the introduction (lines 144 to 147) and the discussion section (lines 810 to 818) of the revised manuscript.

**Comment 4:** I appreciate your discussion of the decision to use listwise deletion of cases with missing data. However, there really is no direct way to test for “missing completely at random” and so I would just report the finding that missing data were not related to your predictors and that is at least consistent with the assumption of “missing at random”. This condition is also consistent with using listwise deletion.

**Response:** We agree that there is no direct way to test whether the data are missing completely at random (MCAR), but this also true for missing at random (MAR) mechanism. According to Rhoads (2012), the empirical data can never provide evidence either for or against the MAR assumption (which implies that missingness may depend on observables but cannot depend on unobservables), and the MCAR assumption (which implies that missingness cannot depend on either observables or unobservables) can be falsified by the data, but it can never be proven true (the data tells us nothing about whether missingness depends on unobservables, but it can tell us whether missingness depends on observables). In other words, MCAR processes require missing data to be MAR (which is not testable), as well as to not depend on observable data (which can be tested). Several tests of MCAR mechanism that exist in the literature (e.g. t-tests or Kruskal Wallis tests for comparison of missing data in the dependent variable across explanatory variables, Little’s test or nonparametric test of homoscedasticity as omnibus tests of MCAR) can only test MCAR relative to MAR alternative hypothesis, while assuming that data are MAR a priori. So, while we cannot directly confirm that the data are MCAR, we can use these tests to examine the patterns of missingness and see if they depend on other observed variables. We have done two of these tests (appropriate for non-normally distributed data) and were very careful to only suggest that that there is no sufficient evidence to reject the assumption of data missing completely at random. In addition, according to the literature (e.g. Little & Rubin, 1987; Rhoads, 2012) listwise deletion requires data to be MCAR in order to produce unbiased estimates of parameters of interest. Because of that, we decided to report in more detail the finding that missing data on criterion variables were not related to our predictors (lines 472 to 481), but also to keep the results of the nonparametric test of homoscedasticity as a proof that the assumption of MCAR cannot be rejected based on the data.

**Comment 5:** In several places you say that you cannot report goodness of fit because the model is saturated. But then you show results with nonsignificant paths deleted (e.g., Figure 1). But you could certainly report the goodness of fit for those results.

**Response:** Thank you for noticing this omission. In two of our path analyses we did estimate only the saturated model because we wanted to interpret all of the model parameters, but did not report or show graphically nonsignificant paths in order to improve the readability and visibility of our results. As the idea was to test the full saturated model, we have now added and described nonsignificant paths both in the path diagram and in the text throughout the results section.

**Comment 6:** Finally, when describing the various ethnic groups, I would try to avoid using the essentialist descriptions whenever possible, i.e., Croats, Serbs, Czechs, Hungarians. For example, on page 15 lines 338-339, you could say “Croatian students responded to items…towards their Serbian peers, and Serbian students toward their Croatian peers.” Making the ethnic classifications predictive rather than attributive encourages an essentialist categorization of ethnic difference, which is something you don’t need to do.

**Response:** We appreciate your concerns regarding the use of appropriate terminology relating to ethnicity and would like to substantiate that we have taken special care in the selection and use of language and ethnic labels during our research with minority participants (including ethnically respectful recruitment strategies, as well as the use of professionally translated study materials upon the request of participants). We have modified the problematic terms in part of the revised manuscript in which we refer to our study participants (lines 344 to 346). However, in the description of our study contexts we kept the “Croat, Serb, Hungarian, Czech and Italian” ethnic labels as these are consistent with data from the Croatian census of population according to ethnicity. We would also like to point out that some of our research measures (which were validated and used in previous research with ethnic minorities) include items containing such labels because they reflect the everyday communication of minority and majority groups in these contexts and are consistent with their common ethnic self-identifications.

We would like to thank you for your thoughtful comments and constructive suggestions which helped us to improve the manuscript quality. We hope that the revised manuscript will now be acceptable for publication in PLOS ONE.

Sincerely,

Lana Pehar
