# Supplementary material for: The role of peer, parental, and school norms in predicting adolescents’ attitudes and behaviours of majority and different minority ethnic groups in Croatia
Source: PLoS One. 2020 Jan 10;15(1):e0227512. doi: 10.1371/journal.pone.0227512 (PMC6953838; doi:10.1371/journal.pone.0227512)
Supplement: Peer review history

# Editor report: Decision Letter 0

**DOI:** 10.1371/journal.pone.0227512.r001

**Authors:** Daniel Romer

29 Jul 2019

PONE-D-19-16051

Normative approach to intergroup relations: The role of peer, parental and school norms about intergroup contact in predicting adolescents’ interethnic attitudes and behaviours

Dear Dr. Pehar,

Thank you for submitting your manuscript to PLOS ONE. After careful consideration, we feel that it has merit but does not fully meet PLOS ONE’s publication criteria as it currently stands. Therefore, we invite you to submit a revised version of the manuscript that addresses the points raised during the review process.

I was unable to obtain any external reviews for your paper and so in the interests of moving your submission along, I have read it myself and have some suggestions for a revision before it can accepted for Plos One. Your paper presents the findings from a survey conducted in 21 elementary and 10 high schools in Croatia focusing on the relations of peer, parental, and school norms with four intergroup outcomes: in-group bias, discrimination, prosocial behavior, and social distance. The literature review identified several questions for which previous research has either been contradictory or lacking. You collected data from both majority and minority youth in the Croatian context with the difference between Croatian and Serbian youth being the most contentious due to the experience of the civil war in the 1990’s. You also collected data in other contexts in which the minority group had better relations with the Croatian majority. Thus, you planned to test differences in the relations between the three types of norms and each outcome depending on differences between majority and minority youth, age, and conflict history. 

The very first question one would raise about the study is the very uneven representation of the various intergroup comparisons in the Table on page 15. I would be skeptical of any ability to study age by context interactions given the very small representation in the Hungarian context and the very uneven age representation in the Czech context. This is the case even before you drop nearly 25% of the sample due to incomplete responses. I would recommend only looking at the Serbian and Italian contexts since they have the largest and most balanced representation of age and minority-majority group status. The other option would be to collapse the various non-Serbian contexts into one, but this would also raise questions about whether this is appropriate given your description of the histories of these groups. 

The second concern is the large variation in the alphas on page 17 for the peer norm and school norm scales for the minority youth. This may affect your conclusions in unknown ways and thus throws your findings regarding differences between the groups into doubt. Perhaps if you drop some of the smaller groups, this will help raise the alphas? As you know, the lower the reliability of a predictor, the less well it will predict outcomes. 

My final concern is the way you show the results. The path diagrams are hard to read (and some are not labelled), and they are inadequate as a description of the findings, especially for interactions. I would encourage you to plot the various significant outcomes of interest in easy to digest graphical format so one can see what the moderation differences look like. It is easy to obtain moderation effects when there are floor or ceiling effects that make the slopes look different, but these may not be very interesting in themselves. So, a reader would want to see the slopes for all three of your interaction predictions whether they are significant or not. 

Finally, I would substantiate that it is acceptable to test for differences between models using the chi-square test associated with robust estimation. I would also provide a stronger rationale for not imputing data for those missing scores. This is an accepted practice if the missingness is sufficiently related to predictors in the dataset. You suggest that this is not the case, but more specific evidence to that effect would be more convincing. You are losing a large proportion of your data, and the dataset is not that large to begin with especially if you drop the two contexts as suggested above.

**********

We would appreciate receiving your revised manuscript by 15 August. When you are ready to submit your revision, log on to https://www.editorialmanager.com/pone/ and select the 'Submissions Needing Revision' folder to locate your manuscript file.

If you would like to make changes to your financial disclosure, please include your updated statement in your cover letter.

To enhance the reproducibility of your results, we recommend that if applicable you deposit your laboratory protocols in protocols.io, where a protocol can be assigned its own identifier (DOI) such that it can be cited independently in the future. For instructions see: http://journals.plos.org/plosone/s/submission-guidelines#loc-laboratory-protocols

Please include the following items when submitting your revised manuscript:

- A rebuttal letter that responds to each point raised by the academic editor. This letter should be uploaded as separate file and labeled 'Response to Reviewers'.
- A marked-up copy of your manuscript that highlights changes made to the original version. This file should be uploaded as separate file and labeled 'Revised Manuscript with Track Changes'.
- An unmarked version of your revised paper without tracked changes. This file should be uploaded as separate file and labeled 'Manuscript'.

Please note while forming your response, if your article is accepted, you may have the opportunity to make the peer review history publicly available. The record will include editor decision letters (with reviews) and your responses to reviewer comments. If eligible, we will contact you to opt in or out.

We look forward to receiving your revised manuscript.

Kind regards,

Daniel Romer

Academic Editor

PLOS ONE

Journal Requirements:

When submitting your revision, we need you to address these additional requirements.

1. Please ensure that your manuscript meets PLOS ONE's style requirements, including those for file naming. The PLOS ONE style templates can be found at

http://www.journals.plos.org/plosone/s/file?id=wjVg/PLOSOne_formatting_sample_main_body.pdf and http://www.journals.plos.org/plosone/s/file?id=ba62/PLOSOne_formatting_sample_title_authors_affiliations.pdf

2. Please include additional information regarding the survey or questionnaire used in the study and ensure that you have provided sufficient details that others could replicate the analyses. For instance, if you developed a questionnaire as part of this study and it is not under a copyright more restrictive than CC-BY, please include a copy, in both the original language and English, as Supporting Information.

3. Please include a new copy of Table 2 in your manuscript; the right side of the current table is not visible in the manuscript page.

Please follow the link for more information: http://blogs.PLOS.org/everyone/2011/05/10/how-to-check-your-manuscript-image-quality-in-editorial-manager/

[Note: HTML markup is below. Please do not edit.]

While revising your submission, please upload your figure files to the Preflight Analysis and Conversion Engine (PACE) digital diagnostic tool, https://pacev2.apexcovantage.com/. PACE helps ensure that figures meet PLOS requirements. To use PACE, you must first register as a user. Registration is free. Then, login and navigate to the UPLOAD tab, where you will find detailed instructions on how to use the tool. If you encounter any issues or have any questions when using PACE, please email us at figures@plos.org. Please note that Supporting Information files do not need this step.

# Author comment: Author response to Decision Letter 0

**DOI:** 10.1371/journal.pone.0227512.r002

29 Nov 2019

Thank you for your review and the opportunity to revise our manuscript „Normative approach to intergroup relations: The role of peer, parental and school norms about intergroup contact in predicting adolescents’ interethnic attitudes and behaviours “. We appreciate the time and effort that you have dedicated to providing valuable feedback on our manuscript and are grateful for your insightful suggestions. We have been able to incorporate changes to reflect most of your suggestions, except the one regarding the proposed sample changes due to reasons we explain in more detail later. The changes were highlighted in the revised manuscript using track changes, as you requested. 

Here is a point-by-point response to your comments and concerns. 

Comment 1: The very first question one would raise about the study is the very uneven representation of the various intergroup comparisons in the Table on page 15. I would be skeptical of any ability to study age by context interactions given the very small representation in the Hungarian context and the very uneven age representation in the Czech context. This is the case even before you drop nearly 25% of the sample due to incomplete responses. I would recommend only looking at the Serbian and Italian contexts since they have the largest and most balanced representation of age and minority-majority group status. The other option would be to collapse the various non-Serbian contexts into one, but this would also raise questions about whether this is appropriate given your description of the histories of these groups.

Response: We agree that studying age by context interactions in this particular sample is difficult due to uneven age representation in specific contexts. However, we think that dropping Czech and Hungarian contexts would lead to substantial loss of important information, as well as to reduced possibility to generalise our findings on context differences related to conflict history to other harmonious intergroup contexts. Also, as you have already pointed out, collapsing all non-Serbian contexts into one would neglect significant differences in histories of intergroup relations between these groups. In order to address the issue that you have pointed out, we decided to avoid all age by context and age by status interactions in moderation analyses because these interactions were never central to our research questions (we are interested in status and contextual differences in the strength of normative effects on intergroup outcomes, not in the strength of age effects). In previous multi-group path analyses for group status and context, we included age as a covariate due to its significant interaction effect with peer norms on social distance, but now we have conducted the multigroup path analyses again without age as a covariate. Changes in the text which reflect differences in results between previous and new analyses are highlighted in sections Moderation effect of group status (lines 643 to 734 in the revised manuscript with tracked changes) and Moderation effects of intergroup context (lines 739 to 805 in the revised manuscript). 

Comment 2: The second concern is the large variation in the alphas on page 17 for the peer norm and school norm scales for the minority youth. This may affect your conclusions in unknown ways and thus throws your findings regarding differences between the groups into doubt. Perhaps if you drop some of the smaller groups, this will help raise the alphas? As you know, the lower the reliability of a predictor, the less well it will predict outcomes.

Response: You have raised an important issue here and we are aware that reliability of a predictor reflects on its possibility to predict the outcome variables. However, due to reasons described in the previous response, we are reluctant to drop some of the smaller groups, especially because we think that doing so would not fix the problem of small and varying reliabilities (e.g. in minority subsample from Croatian-Italian context the reliability of the peer norm subscale is .32 and the reliability of the school norm subscale is .54). Instead, we would like to point out that Cronbach’s alpha, as a measure of reliability, is strongly affected by the length of the scale (the more items in a scale the higher the reliability), depends on the sample being tested (the more heterogenous the sample, the larger the variance of the total scores and the higher the reliability) and is sensitive to even minor deviations from normality which are all potential explanations why Cronbach’s alphas for the peer and school norms subscales in some of our subsamples are low. In addition, Cronbach’s alpha also depends on the content homogeneity of the items and the construct that is being assessed. The items on all our norm subscales were formulated in a way that captures all relevant normative aspects that were identified during the qualitative study. However, factor analysis of the items in the quantitative study resulted in three subscales with different degree of content heterogeneity between the items (parental norm subscale consists of two items with highly similar content, while peer and school norm contain items that tap into somewhat different normative aspects). We explained these potential reasons for low and variable reliabilities in the revised manuscript with tracked changes (lines 387 to 407). Furthermore, we underlined the problem of low and variable reliabilities of our norm subscales in the discussion section of the revised manuscript and have suggested their improvement in future research (lines 937 to 942). 

In addition, we estimated that the way that we have reported Cronbach’s alphas for all study variables in the original manuscript (specifying the range of alphas for majority and minority subsamples from different intergroup contexts) is not relevant for group comparisons that we have presented in our paper. Therefore, in our revised manuscript we have reported Cronbach’s alphas calculated on separate subsamples of minority and majority participants, as well as subsamples of participants from each intergroup context (lines 378 to 380 for peer norms subscale, 380-382 for parental norms subscale, 385-387 for school norms subscale, 419-421 for tendency towards outgroup discrimination scale, 435-437 for prosocial behaviour scale and lines 448 to 450 for social distance scale). 

Comment 3: My final concern is the way you show the results. The path diagrams are hard to read (and some are not labelled), and they are inadequate as a description of the findings, especially for interactions. I would encourage you to plot the various significant outcomes of interest in easy to digest graphical format so one can see what the moderation differences look like. It is easy to obtain moderation effects when there are floor or ceiling effects that make the slopes look different, but these may not be very interesting in themselves. So, a reader would want to see the slopes for all three of your interaction predictions whether they are significant or not.

Response: Thank you for reminding us of how important it is to present the results in a clear and easily interpretable way. We kept only the path diagram for the results on the total sample because we believe that it is relevant and readable and have deleted all figure captions relating to subsequent moderation path diagrams in our revised manuscript. As you suggested, we have presented all of our significant interactions between norm and moderator variables in corresponding interaction plot figures which depict the slopes of predictor variables at different levels of moderator variables and have inserted their figure captions in the revised manuscript (interaction plot figures were submitted as supporting information). We did not graphically present insignificant interactions because we considered that including 36 different interaction plots would overload an already lengthy manuscript. However, if you feel that this is necessary, we will of course include them after the second review. 

Comment 4: Finally, I would substantiate that it is acceptable to test for differences between models using the chi-square test associated with robust estimation.

Response: Thank you for pointing this out. Testing differences between path models with different number of constrained and freed parameters using chi-square difference test is a common way of testing moderation effects in SEM framework. However, when each path model is estimated using Satorra-Bentler scaled chi-square test due to violation of the multivariate normality assumption, the chi-square difference test also needs to be adjusted for the scaling correction. We have substantiated that this is an acceptable procedure with appropriate literature reference (lines 595 to 600 in the revised manuscript).

Comment 5: I would also provide a stronger rationale for not imputing data for those missing scores. This is an accepted practice if the missingness is sufficiently related to predictors in the dataset. You suggest that this is not the case, but more specific evidence to that effect would be more convincing. You are losing a large proportion of your data, and the dataset is not that large to begin with especially if you drop the two contexts as suggested above.

Response: Thank you for this suggestion. We have done a thorough analysis of missing data patterns and found no systematic pattern of nonresponses across the study variables. Specifically, 205 respondents had a missing value on only one of the study variables (59 respondents on social distance, 36 on peer norms, 29 on school norms, 27 on discrimination tendencies, 23 on prosocial behaviour, 20 on parental norms and 11 on ingroup bias), while 129 respondents had some pattern of missing data involving two or more variables. From those, the only unique pattern of missing data found in more than 10 respondents was the one with omitted responses on four variables at the end of the questionnaire and that pattern was found for only 17 respondents. In order to determine whether these data were missing completely at random, we examined if the missing data on criterion variables are related to observed values of predictor variables using R package finalfit. Comparisons of cases with incomplete and complete data on discrimination tendencies variable revealed no significant differences in mean values of peer (p = 0.907), parental (p = .125), and school norms (p = .161) variables. Comparisons on prosocial behaviour variable revealed no significant differences in mean values of peer (p = 0.518), parental (p = .976), and school norms (p = .799) variables. Comparisons on in-group bias variable revealed no significant differences in mean values of peer (p = 0.699), parental (p = .216), and school norms (p = .152) variables. Finally, comparisons on social distance variable also revealed no significant differences in mean values of peer (p = 0.100), parental (p = .132), and school norms (p = .255) variables, showing that the probability of missing data on dependent variables is unrelated to the predictor variables. In addition, we performed the nonparametric test of homoscedasticity developed by Jamshidian and Jalal using R package MissMech. This test examines whether non-normally distributed data has values missing completely at random by testing for homogeneity of covariances between subsets of data having different patterns of missingness (including the one with no missing values). Results of the nonparametric test indicated that there is no sufficient evidence to reject the assumption of data missing completely at random (T = 8.192, p = 0.570). We believe that the results of these analyses provide sufficient evidence to justify listwise deletion of missing data and have summarized them in the revised manuscript (lines 469 to 485). 

Regarding additional journal requirements, we have ensured that our manuscript meets PLOS ONE’s style requirements, including those for file naming. We have also submitted a copy of the questionnaire parts containing all measures used in this research, in both the original language and English as a supporting information file. Finally, we have included a new copy of the Table 2 in our manuscript so that the whole table is now visible in the manuscript page 22.

We hope the revised manuscript will better meet PLOS ONE’s publication criteria, but are happy to consider further revisions, and we thank you for your continued interest in our research.

Attachment Submitted filename: Response to Reviewer.docx Click here for additional data file.

# Editor report: Decision Letter 1

**DOI:** 10.1371/journal.pone.0227512.r003

**Authors:** Daniel Romer

5 Dec 2019

PONE-D-19-16051R1

Normative approach to intergroup relations: The role of peer, parental and school norms about intergroup contact in predicting adolescents' interethnic attitudes and behaviours

PLOS ONE

Dear Dr. Pehar,

Thank you for responding to the concerns raised in the first review. I now mainly have suggestions for making the paper stronger and clearer.

I think your paper will have relevance to educators and others interested in trying to improve interethnic relations in countries such as Croatia. So, I wonder if you might consider a title that makes it clearer that you are studying these issues in a country that has had significant interethnic conflict, something like: The role of peer, parental, and school norms in predicting adolescents’ attitudes and behaviours for both majority and different minority ethnic groups in Croatia. I of course leave this up to you, but I think it will make the abstract a bit more interesting as well.

I also think that the types of schools you are studying will be of interest because the minority youth are segregated from the majority youth in their classes. This would seem to reduce intergroup contact and thus make it more difficult to reduce barriers between the groups. You can’t tell if that matters, but I think it is something that might be worth noting in the discussion.

Related to this point, you seem to downplay the positive role of school norms in your results. On page 35 lines 772-773, you suggest that the significant effects of school norms are due to “a common method factor.” This is confusing because if anything the common variance shared by school norms with the other norms should make it more difficult to detect any effects of school norms. So, the fact that you got relations beyond peer and parental norms for behavioral outcomes is worth highlighting rather than downplaying. Also, this is a factor that is most amenable to intervention, and so it would be worth highlighting it in the introduction as well as the discussion. That is, is there a role for schools despite the segregation of students and the history of conflict to encourage greater intergroup acceptance?

I appreciate your discussion of the decision to use listwise deletion of cases with missing data. However, there really is no direct way to test for “missing completely at random” and so I would just report the finding that missing data were not related to your predictors and that is at least consistent with the assumption of “missing at random.” This condition is also consistent with using listwise deletion.

In several places you say that you cannot report goodness of fit because the model is saturated. But then you show results with nonsignificant paths deleted (e.g., Figure 1). But you could certainly report the goodness of fit for those results.

Finally, when describing the various ethnic groups, I would try to avoid using the essentialist descriptions whenever possible, i.e., Croats, Serbs, Czechs, Hungarians. For example, on page 15 lines 338-339, you could say “Croatian students responded to items…towards their Serbian peers, and Serbian students toward their Croatian peers.” Making the ethnic classifications predictive rather than attributive encourages an essentialist categorization of ethnic difference, which is something you don’t need to do.

When you are ready to submit your revision, log on to https://www.editorialmanager.com/pone/ and select the 'Submissions Needing Revision' folder to locate your manuscript file.

If you would like to make changes to your financial disclosure, please include your updated statement in your cover letter.

To enhance the reproducibility of your results, we recommend that if applicable you deposit your laboratory protocols in protocols.io, where a protocol can be assigned its own identifier (DOI) such that it can be cited independently in the future. For instructions see: http://journals.plos.org/plosone/s/submission-guidelines#loc-laboratory-protocols

Please include the following items when submitting your revised manuscript:

- A rebuttal letter that responds to each point raised by the academic editor and reviewer(s). This letter should be uploaded as separate file and labeled 'Response to Reviewers'.
- A marked-up copy of your manuscript that highlights changes made to the original version. This file should be uploaded as separate file and labeled 'Revised Manuscript with Track Changes'.
- An unmarked version of your revised paper without tracked changes. This file should be uploaded as separate file and labeled 'Manuscript'.

Please note while forming your response, if your article is accepted, you may have the opportunity to make the peer review history publicly available. The record will include editor decision letters (with reviews) and your responses to reviewer comments. If eligible, we will contact you to opt in or out.

We look forward to receiving your revised manuscript.

Kind regards,

Daniel Romer

Academic Editor

PLOS ONE

Journal Requirements:

While revising your submission, please upload your figure files to the Preflight Analysis and Conversion Engine (PACE) digital diagnostic tool, https://pacev2.apexcovantage.com/. PACE helps ensure that figures meet PLOS requirements. To use PACE, you must first register as a user. Registration is free. Then, login and navigate to the UPLOAD tab, where you will find detailed instructions on how to use the tool. If you encounter any issues or have any questions when using PACE, please email us at figures@plos.org. Please note that Supporting Information files do not need this step.

# Author comment: Author response to Decision Letter 1

**DOI:** 10.1371/journal.pone.0227512.r004

19 Dec 2019

Here is a point-by-point response to your comments and concerns. 

Comment 1: I think your paper will have relevance to educators and others interested in trying to improve interethnic relations in countries such as Croatia. So, I wonder if you might consider a title that makes it clearer that you are studying these issues in a country that has had significant interethnic conflict, something like: The role of peer, parental, and school norms in predicting adolescents’ attitudes and behaviours for both majority and different minority ethnic groups in Croatia. I of course leave this up to you, but I think it will make the abstract a bit more interesting as well.

Response: Thank you for this excellent observation, we have changed the title of our manuscript according to your proposal on first page of the manuscript (lines 4 to 5 in the revised manuscript). 

Comment 2: I also think that the types of schools you are studying will be of interest because the minority youth are segregated from the majority youth in their classes. This would seem to reduce intergroup contact and thus make it more difficult to reduce barriers between the groups. You can’t tell if that matters, but I think it is something that might be worth noting in the discussion.

Response: We agree with this and have incorporated a comment relating to specific school contexts in our study in the discussion section of the revised manuscript (lines 790 to 797).

Comment 3: Related to this point, you seem to downplay the positive role of school norms in your results. On page 35 lines 772-773, you suggest that the significant effects of school norms are due to “a common method factor.” This is confusing because if anything the common variance shared by school norms with the other norms should make it more difficult to detect any effects of school norms. So, the fact that you got relations beyond peer and parental norms for behavioural outcomes is worth highlighting rather than downplaying. Also, this is a factor that is most amenable to intervention, and so it would be worth highlighting it in the introduction as well as the discussion. That is, is there a role for schools despite the segregation of students and the history of conflict to encourage greater intergroup acceptance?

Response: We appreciate your concern regarding our explanation of the school norms effect. However, we did not want to say that the significant effect of the school norms is due to a common method factor shared by different measures of contacts norms, but instead due to common variance shared by our measure of school norms and measures of discrimination tendencies and prosocial behaviour (all three measures include items related to the school context). We rephrased this part in our revised manuscript to make it clearer, as well as to avoid the “common method factor” implications (lines 818 to 823). In addition, we agree with your observation that we did not sufficiently emphasize the positive role of school norms in our results. Accordingly, we highlighted the potential of school norms in improving intergroup relations, both in the introduction (lines 144 to 147) and the discussion section (lines 810 to 818) of the revised manuscript. 

Comment 4: I appreciate your discussion of the decision to use listwise deletion of cases with missing data. However, there really is no direct way to test for “missing completely at random” and so I would just report the finding that missing data were not related to your predictors and that is at least consistent with the assumption of “missing at random”. This condition is also consistent with using listwise deletion.

Response: We agree that there is no direct way to test whether the data are missing completely at random (MCAR), but this also true for missing at random (MAR) mechanism. According to Rhoads (2012), the empirical data can never provide evidence either for or against the MAR assumption (which implies that missingness may depend on observables but cannot depend on unobservables), and the MCAR assumption (which implies that missingness cannot depend on either observables or unobservables) can be falsified by the data, but it can never be proven true (the data tells us nothing about whether missingness depends on unobservables, but it can tell us whether missingness depends on observables). In other words, MCAR processes require missing data to be MAR (which is not testable), as well as to not depend on observable data (which can be tested). Several tests of MCAR mechanism that exist in the literature (e.g. t-tests or Kruskal Wallis tests for comparison of missing data in the dependent variable across explanatory variables, Little’s test or nonparametric test of homoscedasticity as omnibus tests of MCAR) can only test MCAR relative to MAR alternative hypothesis, while assuming that data are MAR a priori. So, while we cannot directly confirm that the data are MCAR, we can use these tests to examine the patterns of missingness and see if they depend on other observed variables. We have done two of these tests (appropriate for non-normally distributed data) and were very careful to only suggest that that there is no sufficient evidence to reject the assumption of data missing completely at random. In addition, according to the literature (e.g. Little & Rubin, 1987; Rhoads, 2012) listwise deletion requires data to be MCAR in order to produce unbiased estimates of parameters of interest. Because of that, we decided to report in more detail the finding that missing data on criterion variables were not related to our predictors (lines 472 to 481), but also to keep the results of the nonparametric test of homoscedasticity as a proof that the assumption of MCAR cannot be rejected based on the data. 

Comment 5: In several places you say that you cannot report goodness of fit because the model is saturated. But then you show results with nonsignificant paths deleted (e.g., Figure 1). But you could certainly report the goodness of fit for those results.

Response: Thank you for noticing this omission. In two of our path analyses we did estimate only the saturated model because we wanted to interpret all of the model parameters, but did not report or show graphically nonsignificant paths in order to improve the readability and visibility of our results. As the idea was to test the full saturated model, we have now added and described nonsignificant paths both in the path diagram and in the text throughout the results section. 

Comment 6: Finally, when describing the various ethnic groups, I would try to avoid using the essentialist descriptions whenever possible, i.e., Croats, Serbs, Czechs, Hungarians. For example, on page 15 lines 338-339, you could say “Croatian students responded to items…towards their Serbian peers, and Serbian students toward their Croatian peers.” Making the ethnic classifications predictive rather than attributive encourages an essentialist categorization of ethnic difference, which is something you don’t need to do.

Response: We appreciate your concerns regarding the use of appropriate terminology relating to ethnicity and would like to substantiate that we have taken special care in the selection and use of language and ethnic labels during our research with minority participants (including ethnically respectful recruitment strategies, as well as the use of professionally translated study materials upon the request of participants). We have modified the problematic terms in part of the revised manuscript in which we refer to our study participants (lines 344 to 346). However, in the description of our study contexts we kept the “Croat, Serb, Hungarian, Czech and Italian” ethnic labels as these are consistent with data from the Croatian census of population according to ethnicity. We would also like to point out that some of our research measures (which were validated and used in previous research with ethnic minorities) include items containing such labels because they reflect the everyday communication of minority and majority groups in these contexts and are consistent with their common ethnic self-identifications.

Attachment Submitted filename: Response to reviewer.docx Click here for additional data file.

# Editor report: Decision Letter 2

**DOI:** 10.1371/journal.pone.0227512.r005

**Authors:** Daniel Romer

20 Dec 2019

The role of peer, parental, and school norms in predicting adolescents’ attitudes and behaviours of majority and different minority ethnic groups in Croatia

PONE-D-19-16051R2

Dear Dr. Pehar,

We are pleased to inform you that your manuscript has been judged scientifically suitable for publication and will be formally accepted for publication once it complies with all outstanding technical requirements.

Within one week, you will receive an e-mail containing information on the amendments required prior to publication. When all required modifications have been addressed, you will receive a formal acceptance letter and your manuscript will proceed to our production department and be scheduled for publication.

Shortly after the formal acceptance letter is sent, an invoice for payment will follow. To ensure an efficient production and billing process, please log into Editorial Manager at https://www.editorialmanager.com/pone/, click the "Update My Information" link at the top of the page, and update your user information. If you have any billing related questions, please contact our Author Billing department directly at authorbilling@plos.org.

If your institution or institutions have a press office, please notify them about your upcoming paper to enable them to help maximize its impact. If they will be preparing press materials for this manuscript, you must inform our press team as soon as possible and no later than 48 hours after receiving the formal acceptance. Your manuscript will remain under strict press embargo until 2 pm Eastern Time on the date of publication. For more information, please contact onepress@plos.org.

With kind regards,

Daniel Romer

Academic Editor

PLOS ONE

Additional Editor Comments:

Thank you for making the changes to the paper. I have one suggestion for wording. On line 801-802 page 36, it would be clearer to say "did not predict attitudinal measures of bias..." Your wording is confusing, at least to me.

Although I do not insist on the issue of MAR vs. MCAR, it is now generally recognized that data that are MAR can be analyzed with listwise deletion without bias. So, although it is unnecessary to argue that your data are MCAR, there is no harm making that claim (even if it is incorrect).

# Editor report: Acceptance letter

**DOI:** 10.1371/journal.pone.0227512.r006

**Authors:** Daniel Romer

27 Dec 2019

PONE-D-19-16051R2 

The role of peer, parental, and school norms in predicting adolescents’ attitudes and behaviours of majority and different minority ethnic groups in Croatia 

Dear Dr. Pehar:

I am pleased to inform you that your manuscript has been deemed suitable for publication in PLOS ONE. Congratulations! Your manuscript is now with our production department. 

If your institution or institutions have a press office, please notify them about your upcoming paper at this point, to enable them to help maximize its impact. If they will be preparing press materials for this manuscript, please inform our press team within the next 48 hours. Your manuscript will remain under strict press embargo until 2 pm Eastern Time on the date of publication. For more information please contact onepress@plos.org.

For any other questions or concerns, please email plosone@plos.org. 

Thank you for submitting your work to PLOS ONE.

With kind regards,

PLOS ONE Editorial Office Staff

on behalf of

Dr. Daniel Romer 

Academic Editor

PLOS ONE
